# The evolution of farnesoid X, vitamin D, and pregnane X receptors: insights from the green-spotted pufferfish (*Tetraodon nigriviridis*) and other non-mammalian species

**DOI:** 10.1186/1471-2091-12-5

**Published:** 2011-02-03

**Authors:** Matthew D Krasowski, Ni Ai, Lee R Hagey, Erin M Kollitz, Seth W Kullman, Erica J Reschly, Sean Ekins

**Affiliations:** 1 Department of Pathology, University of Iowa Hospitals and Clinics, Iowa City, IA, 52242, USA; 2 Department of Pathology, University of Pittsburgh, Pittsburgh, PA, 15261 USA; 3 Department of Pharmacology, Robert Wood Johnson Medical School, University of Medicine & Dentistry of New Jersey, Piscataway, NJ 08854, USA; 4 Department of Medicine, University of California - San Diego, La Jolla, CA, 92093 USA; 5 Department of Environmental and Molecular Toxicology, North Carolina State University, Raleigh, NC 27695, USA; 6 Collaborations in Chemistry, Jenkintown, PA 19046, USA; 7 Department of Pharmaceutical Sciences, University of Maryland, Baltimore, MD 21201, USA

## Abstract

**Background:**

The farnesoid X receptor (FXR), pregnane X receptor (PXR), and vitamin D receptor (VDR) are three closely related nuclear hormone receptors in the NR1H and 1I subfamilies that share the property of being activated by bile salts. Bile salts vary significantly in structure across vertebrate species, suggesting that receptors binding these molecules may show adaptive evolutionary changes in response. We have previously shown that FXRs from the sea lamprey (*Petromyzon marinus*) and zebrafish (*Danio rerio*) are activated by planar bile alcohols found in these two species. In this report, we characterize FXR, PXR, and VDR from the green-spotted pufferfish (*Tetraodon nigriviridis*), an actinopterygian fish that unlike the zebrafish has a bile salt profile similar to humans. We utilize homology modelling, docking, and pharmacophore studies to understand the structural features of the *Tetraodon *receptors.

**Results:**

*Tetraodon *FXR has a ligand selectivity profile very similar to human FXR, with strong activation by the synthetic ligand GW4064 and by the primary bile acid chenodeoxycholic acid. Homology modelling and docking studies suggest a ligand-binding pocket architecture more similar to human and rat FXRs than to lamprey or zebrafish FXRs. *Tetraodon *PXR was activated by a variety of bile acids and steroids, although not by the larger synthetic ligands that activate human PXR such as rifampicin. Homology modelling predicts a larger ligand-binding cavity than zebrafish PXR. We also demonstrate that VDRs from the pufferfish and Japanese medaka were activated by small secondary bile acids such as lithocholic acid, whereas the African clawed frog VDR was not.

**Conclusions:**

Our studies provide further evidence of the relationship between both FXR, PXR, and VDR ligand selectivity and cross-species variation in bile salt profiles. Zebrafish and green-spotted pufferfish provide a clear contrast in having markedly different primary bile salt profiles (planar bile alcohols for zebrafish and sterically bent bile acids for the pufferfish) and receptor selectivity that matches these differences in endogenous ligands. Our observations to date present an integrated picture of the co-evolution of bile salt structure and changes in the binding pockets of three nuclear hormone receptors across the species studied.

## Background

Nuclear hormone receptors (NHRs) are transcription factors that work in concert with co-activators and co-repressors to regulate gene expression [1,2]. Most of the NHRs in vertebrates are ligand-activated, although some NHRs function in a ligand-independent manner. Examples of ligands for NHRs include a range of endogenous compounds such as bile acids, retinoids, steroid hormones, thyroid hormone, and vitamin D. A few NHRs, such as the pair of xenobiotic sensors, pregnane X receptor (PXR; NR1I2; also known as steroid and xenobiotic receptor or SXR) and constitutive androstane receptor (CAR; NR1I3), are activated by structurally diverse exogenous ligands. NHRs share a conserved domain structure, which includes, from N-terminus to C-terminus, a modulatory A/B domain, the DNA-binding domain (DBD; C domain), the 'hinge' D domain, the ligand-binding domain (LBD; E domain), and a variable C-terminal F domain that is absent in some NHRs [2,3].

NHRs show varying degrees of sequence conservation across vertebrate species in the LBD that likely reflects, at least in part, cross-species variation in the physiologically important ligands. The xenobiotic sensors PXR and CAR show extensive cross-species amino acid sequence divergence across vertebrate species [4,5] in addition to evidence of positive (Darwinian) selection in the LBD from phylogenetic analyses [6,7]. Cross-species differences in xenobiotic ligands are a possible driving force for changes in the LBD sequence and structure. We and others have also provided data consistent with the hypothesis that the structure of the LBD of NHRs in the NR1 H and NR1I subfamilies may have co-evolved with the endogenous ligands in some species in evolution [4,6-12].

**Figure 1:**
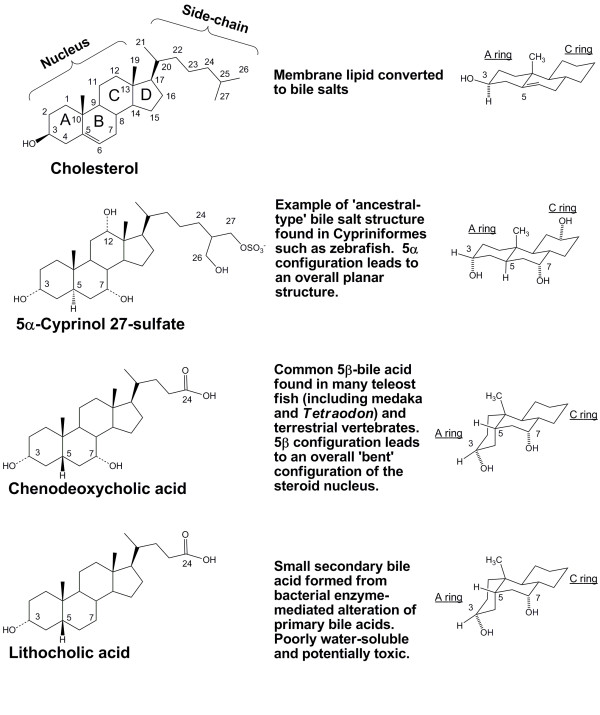
**Representative bile salts and their structures**. All bile salts are derived from cholesterol (topmost structure), illustrated with the carbon atoms numbered and the steroid rings labelled A, B, C, and D. Jawless fish, lobe-finned fish, and a limited number of actinopterygian fish use 5α bile alcohols such as 5α-cyprinol-27-sulfate that have an overall planar and extended structure of the steroid rings (see representation of A, B, and C rings on the right side). Most actinopterygian fish (including medaka and *Tetraodon nigrivirdis*) use 5β bile acids that have an overall bent structure of the steroid rings. One of the two most common primary salts in mammals is chenodeoxycholic acid (CDCA), the stem C_24 _bile acid that has the basic 3α,7α-dihydroxylation pattern. Lithocholic acid is one of the smallest naturally occurring bile acids and results from bacterial enzyme-mediated deconjugation and dehydroxylation of primary bile acids. The sodium and calcium salts of lithocholic acid have very low solubility at body temperature. Additionally, lithocholic acid is toxic in humans and other mammals.

Bile salts are one class of NHR ligands that show substantial cross-species differences in chemical structure [13]. Bile salts are amphipathic, water-soluble end-metabolites of cholesterol that facilitate intestinal absorption of lipids, enhance proteolytic cleavage of dietary proteins, and exert antimicrobial activity in the small intestine [14,15]. Bile salts exhibit significant structural diversity across vertebrate species [13,16-18] and include *bile alcohols *(which have a hydroxyl group on the terminal carbon atom of the side-chain) and *bile acids *(which have a carboxylic acid group on the side-chain) (Figure 1) [15]. Primary bile salts are defined as those synthesized by the liver, which is accomplished by a complicated biosynthetic pathway that starts with cholesterol [19,20].

Secondary bile salts result from modification of primary bile salts by host bacteria in the intestine [15]. Common alterations in bile acids catalyzed by enzymes from anaerobic bacteria in the intestinal tract include deconjugation and dehydroxylation, with one possible end result being unconjugated and poorly water-soluble bile acids such as lithocholic acid (3α-hydroxy-5β-cholan-24-oic acid; LCA), a compound known to be toxic to mammalian species including humans [15,21]. There have been few studies of intestinal bile salts in animals that utilize bile alcohols, but one study of the Asiatic carp (*Cyprinus carpio*) showed that the bile alcohol sulfates synthesized in the liver of this species did not undergo hydrolysis in the intestinal tract and thus did not generate potentially toxic unconjugated secondary bile alcohols [22].

Variation in the stereochemistry of the junction between rings A and B of the bile salt steroid nucleus determines whether bile salts have a flat (planar) or a 'bent' orientation. Based on surveys of the bile salt composition in 1153 vertebrate species, we have proposed that 5α (planar) bile alcohols are the likely ancestral ligands (the paleomorphic state) while 5β (bent) bile acids are the derived (apomorphic) phenotype [13,23-25]. Common 5β-bile acids include chenodeoxycholic acid (CDCA; 3α,7α-dihydroxy-5β-cholan-24-oic acid) and cholic acid (CA; 3α,7α,12α-trihydroxy-5β-cholan-24-oic acid), the two dominant primary bile acids in humans and other mammals (Figure 1) [13,24].

PXRs are activated by a structurally diverse array of endogenous and exogenous molecules that includes bile salts, steroid hormones, prescription medications, herbal drugs, and endocrine disruptors [26,27]. PXR regulates the transcription of enzymes and transporters involved in the metabolism and elimination of potentially harmful compounds, including sulfation of toxic bile acids [28]. Previous studies have shown substantial cross-species differences in PXR ligand specificity, including in the selectivity for bile salts [4,7,8]. Mammalian PXRs are activated by a broad range of bile salt structures (both ancestral and evolutionarily derived), while chicken (*Gallus gallus*) and zebrafish (*Danio rerio*) PXRs are activated by a structurally narrow range of bile salts [4,6,7,9,29]. We and others have proposed that the evolution of PXRs has been driven by at least two factors: adaptation to evolutionary changes in bile salt (and perhaps other endogenous molecular) structure and function as a xenobiotic sensor [4,7,9,12]. Compared with published X-ray crystallographic structures of human PXR [30-35], a homology model of the zebrafish PXR is predicted to have a smaller ligand-binding pocket (LBP) than human PXR, with a flat ligand-binding surface well-suited to binding the planar 5α-bile alcohols that are the primary bile salts of zebrafish and other cypriniform fish [9].

FXR serves as the major transcriptional regulator of bile salt synthesis, in part by controlling the expression of cytochrome P450 (CYP) 7A1, the rate-limiting enzyme in the synthetic pathway [36]. Mammalian FXRs are activated best by primary bile acids CDCA and CA [37-39]. FXR is typically expressed at high levels in the liver, intestine, kidney, and adrenal glands. A second FXR, termed FXRβ (NR1H5), is found in some animal species (although it is a pseudogene in the genome of some mammals such as humans and other primates) but does not appear to be involved with bile salt binding or regulation [40]. Throughout this manuscript, FXR refers to NR1H4, or what might be termed FXRα in species possessing two FXRs.

The African clawed frog (*Xenopus laevis*) expresses an unusual FXR (also termed FOR, FXR-like orphan receptor) that has a 33 amino acid insert, not found in mammalian FXRs, in helix 7 of the ligand-binding domain (LBD) [41]. We have previously demonstrated that sea lamprey (*Petromyzon marinus*), zebrafish, and African clawed frog FXRs are activated by bile alcohols [9]. Sea lamprey and zebrafish FXRs are selective for planar 5α bile alcohols. The *Xenopus *FXR isoform 1 was activated by 5α- and 5β-bile alcohols (paralleling the complex bile salt profile of this amphibian [23]) but was poorly activated by bile acids [9,41].

The vitamin D receptor (VDR; NR1I1) is known to mediate the action of 1,25-dihydroxyvitamin D (calcitriol), a hormone whose 'classic' function is to regulate calcium and phosphorus homeostasis. However, VDRs are now known to be involved in a wide range of physiological processes including immune system modulation, skin development, and regulation of the metabolism of toxic compounds [42-45]. Two VDR genes have been found in the genome of many actinopterygian fish (a likely by-product of whole genome duplication prior to actinopterygian fish radiation) [46-48], with VDRα and VDRβ both shown to be functional in the Japanese medaka (*Oryzias latipes*) [49]. Mammalian VDRs are activated at low affinity by a narrow range of 5β-bile acids, particularly the toxic secondary bile acid LCA and its derivatives [7,50-52]. VDR activation in the intestine has been shown to upregulate the expression of enzymes (e.g., CYP3A) that can metabolize and reduce the toxicity of LCA [52-56]. We previously determined that the VDR from the sea lamprey (*Petromyzon marinus*) was insensitive to activation by a wide array of bile salts, including bile acids and bile alcohols with a range of substituents. We proposed the hypothesis that activation of VDRs by bile acids is a 'derived' trait, possibly as an adaptation to evolutionary changes in bile salt pathways and vertebrate physiology that allow for generation of toxic secondary bile acids [6,7,9,10].

In this report, we characterize the ligand selectivity of FXR, VDR, and PXR from the green-spotted pufferfish (*Tetraodon nigriviridis*), an actinopterygian fish that synthesizes mainly the 5β-bile acids CA and CDCA [23], thereby having a bile salt profile similar to most mammals. We also cloned and characterized VDRs from three additional non-mammalian species: medaka, African clawed frog, and chicken (*Gallus gallus*). In terms of primary bile salts, the medaka synthesizes a mixture of 5β C_24 _and C_27 _bile acids [57,58]. The African clawed frog has a complicated mixture of C_26 _and C_27 _bile alcohols, C_24 _bile acids, and C_27 _bile acids in its bile [9,23]. The chicken synthesizes mainly CA and CDCA as primary bile salts [25].

We generated homology models of *Tetraodon *FXR (tnFXR) and PXR (tnPXR) using rat FXR [59] and human PXR [32] crystallographic structures, respectively, as templates. We then performed computational ligand docking experiments to these receptors. This allowed for comparison to the structures of the corresponding mammalian receptors, helping to formulate hypotheses to rationalize the structural changes that have occurred during receptor evolution. Additionally, we build on our previous docking studies with bile acids and human VDR (hVDR) and expand structure-activity series of bile acids at this receptor. Combining information from docking experiments and cross-species sequence comparisons, we generated site-directed mutations and chimeras to better understand the molecular determinants of bile salt activation of VDRs.

## Results

### Bile salt profile of *Tetraodon nigriviridis*

We previously reported a survey of the biliary bile salts of 213 fish species from 38 different orders [23]. Included in this survey were six species from Tetraodontiformes, an order of actinopterygian fish that includes *Tetraodon nigriviridis*. The primary bile salts from all six species of Tetraodontiformes examined were the common 5β-bile acids CA and CDCA. This is illustrated in Additional file 1 by high-performance liquid chromatography (HPLC) and electrospray ionization-tandem mass spectrometry (ESI/MS/MS) (Additional file 1: figures S1A-S1C). The primary bile salt pool of *Tetraodon *is quite similar to that of many mammals (including humans) and birds [13,16,17,24,25].

### Ligand selectivity of the *Tetraodon *FXR

**Figure 2:**
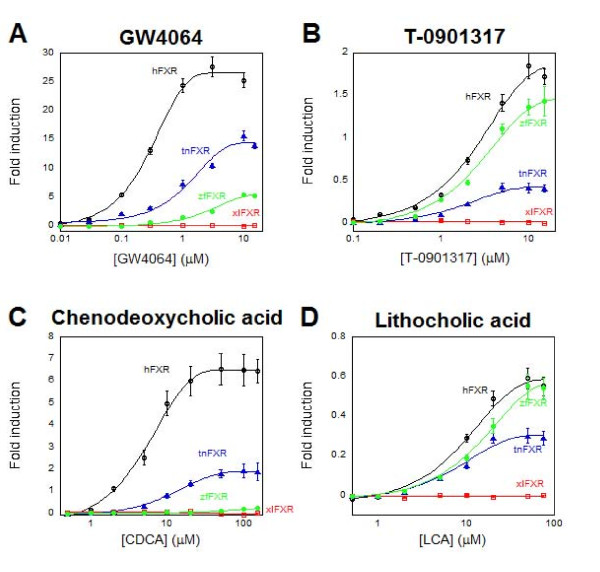
**FXR concentration-response curves**. Ligand activation of FXRs from different species. A) The synthetic mammalian FXR activator GW4064 activates human FXR (hFXR) with high efficacy and submicromolar potency. GW4064 also activates *Tetraodon *FXR (tnFXR) and zebrafish FXR (zfFXR) but not *Xenopus laevis *FXR(xlFXR). B) T-0901317 activates hFXR, zfFXR, and tnFXR (although with lower efficacy compared to GW4064) but not xlFXR. C) The primary bile acid chenodeoxycholic acid (CDCA) activates hFXR and tnFXR but not zfFXR or xlFXR. D) The secondary bile acid lithocholic acid (LCA) activates hFXR, tnFXR, and zfFXR, but not xlFXR. The ordinate indicates fold induction compared to vehicle control in luciferase-based assay. Note that the scale of the ordinate is different in A) through D).

**Figure 3:**
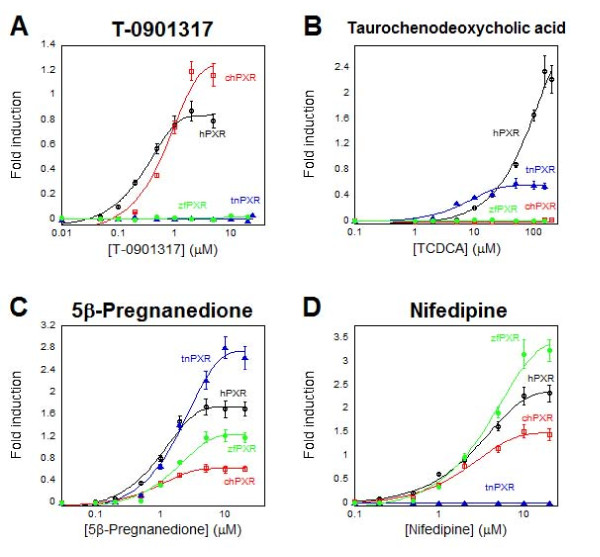
**PXR concentration-response curves**. Ligand activation of PXRs from different species. A) T-0901317 activates human PXR (hPXR) and chicken PXR (chPXR) but not *Tetraodon *PXR (tnPXR) or zebrafish PXR (zfPXR). B) The primary conjugated bile acid taurochenodeoxycholic acid (TCDCA) activates hPXR and tnPXR but not chPXR or zfPXR. C) The steroid 5β-pregnane-3,20-dione (5β-pregnanedione) activates all four PXRs shown. D) Nifedipine activates hPXR, chPXR, and zfPXR, but not tnPXR. The ordinate indicates fold induction compared to vehicle control in luciferase-based assay. Note that the scale of the ordinate is different in A) through D).

Analysis of the draft genome of *Tetraodon nigriviridis *revealed a single putative ortholog to human FXRα (hFXR) and another single putative ortholog to human PXR (hPXR). We did not find any evidence of an ortholog to FXRβ in the *Tetraodon *genome. The LBDs of tnFXR and tnPXR were cloned and functionally expressed as GAL4/LBD chimeras (concentration-response data in Figure 2 and 3). tnFXR was activated most strongly by GW4064 (Figure 2A), a synthetic ligand known to activate mammalian FXRs with high efficacy [60], and activated weakly by T-0901317 (Figure 2B), an agonist of mammalian liver X receptors (LXRs; NR1H2 and NR1H3), FXRs, and PXRs [34,61,62]. Conjugated and unconjugated 5β bile acids also activated tnFXR, including CDCA, tauro-CDCA, 3-oxo-LCA, and LCA (Figure 2C,D; Additional file 2). The EC_50 _of bile acids for activation of tnFXR ranged from 8.8 to 37.6 μM, with maximal effects varying from 15-61% of that produced by GW4064 (which served as the reference compound for comparisons of maximal effect or efficacy). The planar bile alcohol 5α-cyprinol sulfate (the main bile salt of cypriniform fish but only a very minor component of *Tetraodon *bile salts) weakly activated tnFXR. A 76-compound library of known NHR ligands was screened for additional activators of tnFXR. This screening identified three activators of tnFXR in addition to bile acids which included two farnesol derivatives (farnesol and *S*-farnesyl-*L*-cysteine methyl ester) and 6-formylindolo-[3,2-*b*]-carbazole.

### Ligand selectivity of the *Tetraodon *PXR

With respect to bile salts, tnPXR had similar structure-activity relationships to tnFXR, namely activation by 5β-bile acids with one or two hydroxyl groups on the nuclear rings (Figure 3; Additional file 2). Unlike tnFXR, tnPXR was not activated by GW4064 or farnesol compounds. tnPXR was also activated by a variety of androstane, estrane, and pregnane steroids, similar to PXRs from mammals, chicken, and zebrafish (Figure 3C) [4]. tnPXR was not activated by several xenobiotics known to be agonists of mammalian PXRs including hyperforin (pharmacologically active component of the herbal antidepressant St. John's wort), nifedipine (Figure 3D), rifampicin, and SR12813, but was activated by *n*-butyl 4-amino benzoate, an agonist of *Xenopus laevis *PXRs [4,63] (Additional file 2).

### Structure-activity relationship of bile acids for activation of human and mouse VDRs

**Figure 4:**
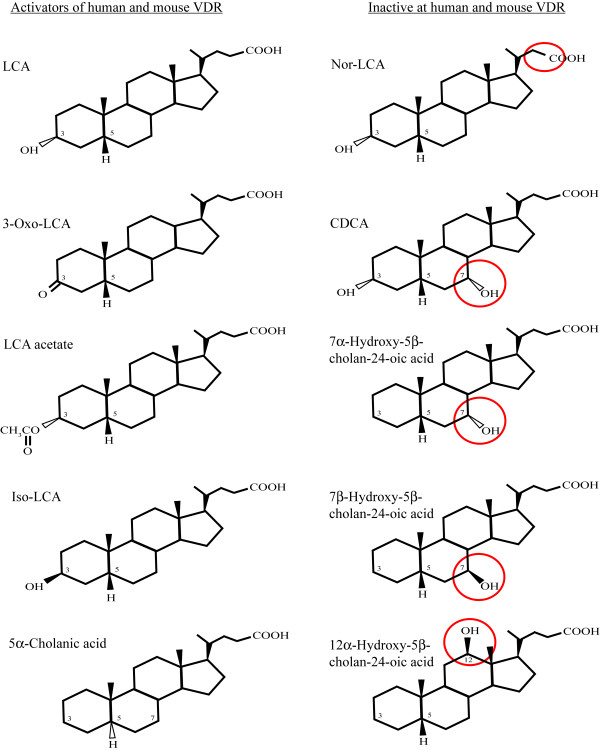
**Structure-activity for human and mouse VDR**. Structure-activity data for bile acid activation of human and mouse VDRs. The bile acids that activate human and mouse VDRs are small, hydrophobic bile acids that are related to lithocholic acid (LCA) and have either a hydroxyl or oxo group at C-3 (LCA, 3-oxo-LCA, LCA acetate, iso-LCA) or have unsubstituted steroid ring (5α-cholanic acid). Addition of a 7α-hydroxy group to LCA (resulting in the bile acid CDCA) or even single steroid ring substitutions at C-7 or C-12 result in bile acids that do not activate the VDRs. Nor-LCA, which has a shortened bile acid side-chain, is also inactive.

Expanding on previous studies from our group [6,7,10] and others [50,52], we determined the structure-activity relationships of naturally occurring and synthetic bile acids for transactivation of GAL4/LBD constructs of hVDR and mouse VDR (mVDR). LCA and derivatives (e.g., 3-oxo-LCA, LCA acetate) activated both hVDR and mVDR (Figure 4; Additional file 3). Iso-LCA, with a single 3β-hydroxy group on the steroid rings (as opposed to 3α-hydroxy of LCA), weakly activated hVDR and mVDR. In contrast, naturally occurring 5β-bile acids with two or three hydroxyl groups on the nuclear rings were inactive including CDCA (3α,7α-dihydroxy-5β-cholan-24-oic acid), DCA (3α,12α-dihydroxy), and CA (3α,7α,12α-trihydroxy). We also tested three 5β-bile acids not known to occur naturally in bile that have a single hydroxyl substituent on the steroid rings on a carbon other than C-3 (7α-hydroxy, 7β-hydroxy, and 12α-hydroxy-5β-cholan-24-oic acids), as well as unsubstituted 5β-cholanic acid (no hydroxyl groups on any of the steroid rings). All four of these bile acids were inactive with respect to activation of hVDR and mVDR. Thus, bile acids with hydroxyl groups at the C-7 or C-12 position are unfavourable for activation of hVDR (Figure 4). Unsubstituted 5α-cholanic acid, which would have an overall planar orientation of the steroid rings, weakly activated hVDR and mVDR. Two 5α-cholanic acid derivatives (3β-hydroxy and 3-oxo) were inactive (Additional file 3).

### Activation of non-mammalian VDRs by bile salts

**Figure 5:**
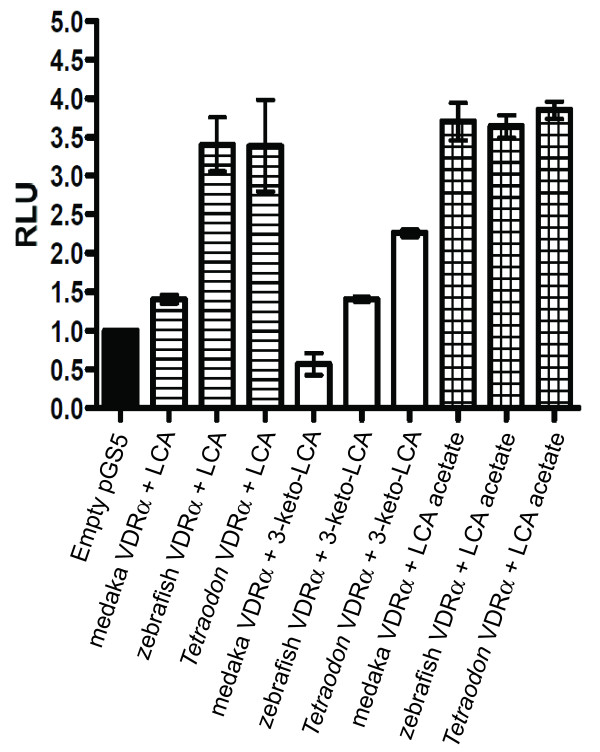
**Transactivation of full-length teleost VDRs**. HepG2 cells were transiently transfected with pRL-CMV, XREM-Luc and either medaka VDRα-pSG5, zebrafish VDRα-pSG5, or *Tetraodon *VDRα-pSG5 as described in Methods. Cells were exposed to 100 μM of either lithocholic acid (LCA), 3-keto-LCA, or LCA acetate for 24 hours. VDR response was measured via dual-luciferase assays. Data is represented as the mean fold induction normalized to control (DMSO) ± SEM.

**Figure 6:**
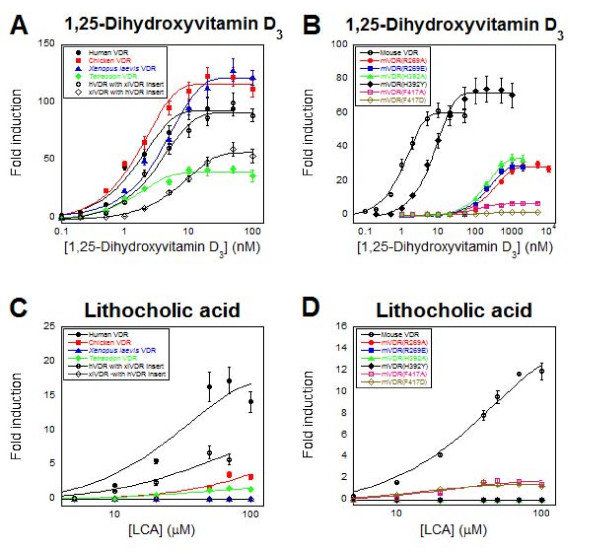
**VDR concentration-response curves**. Ligand activation of VDRs from different species, chimeric human-frog VDRs, and mouse VDR site-directed mutants. A) The plot shows activation of human, chicken, *Xenopus laevis*, and *Tetraodon *VDRs by 1,25-dihydroxyvitamin D_3_. Also shown are activation data for a chimeric receptor that is mostly human VDR (hVDR) with the H1-H3 insert replaced by the corresponding sequence from *Xenopus laevis *VDR (xlVDR) and the converse chimeric receptor that is mostly xlVDR with the H1-H3 insert replaced by the corresponding sequence from hVDR. B) The plot shows activation of mouse VDR (mVDR) and six site-directed mutants of mVDR (R269A, R269E, H392A, H392Y, F417A, and F417D) by 1,25-dihydroxyvitamin D_3_. C) Lithocholic acid (LCA) activates human, chicken, and *Tetraodon *VDRs, as well as the chimeric receptor hVDR with xlVDR h1-H3 insert, but does not activate xlVDR and the chimeric receptor xlVDR with the hVDR H1-H3 insert. D) Lithocholic acid strongly activates mVDR, weakly activates the site-directed mutants F417A and F417D, and does not activate R269A, R269E, H392A, and H392Y. The ordinate indicates fold induction compared to vehicle control in luciferase-based assay. Note that the scale of the ordinate is different in A) through D).

The African clawed frog VDR (*Xenopus laevis *VDR; xlVDR) was not activated by any bile salts tested, including bile alcohols. In contrast, chicken VDR (chVDR), medaka VDRα (olVDRα), *Tetraodon *VDRα (tnVDR), and zebrafish VDRα (zfVDRα) were each activated by LCA and/or its derivatives (3-keto-LCA and LCA acetate) but not by bile acids with two or more hydroxyl groups such as CDCA, DCA, or CA (Figure 5 and 6; Additional file 3). The efficacies of LCA, 3-oxo-LCA, and LCA acetate (in comparison to 1,25α-dihydroxyvitamin D_3_) for activation of chicken, medaka, *Tetraodon*, zebafish VDRs were lower than for hVDR and mVDR (Figure 6; Additional file 3).

### Structure-directed mutagenesis experiments

We previously used molecular modelling computational docking studies to understand the structural basis of bile acid activation of hVDR and mVDR [9]. These studies predicted an electrostatic interaction between Arg-274 (hVDR numbering) and the bile acid side-chain, and a hydrogen bond between the 3α-hydroxyl group of LCA and His-397 in helix 11 (note corresponding residue numbers are 5 lower for mVDR; e.g., Arg-269 in mVDR is equivalent to Arg-274 in hVDR). This hydrogen bonding brings LCA close to the activation helix 12 where LCA forms hydrophobic contacts with Val-398 and Phe-422 that would stabilize the helix in the optimal orientation for coactivator binding. Site-directed mutagenesis by Adachi *et al. *supported this conclusion and indicated that alteration on this Arg residue of hVDR (e.g., Arg274Leu) significantly disrupted the receptor response to LCA [51]. Additional file 4 displays the surface around the ligand binding pocket of hVDR, showing that it is predominantly hydrophobic in the middle with more polar features on its ends.

We next performed site-directed mutagenesis experiments to confirm the docking model of the bile acid to VDR, and to try to rationalize the cross-species differences in activation of VDR by bile salts. These mutations were performed in mVDR, which generally has higher maximal activation by bile acids but shows a similar selectivity for bile acids to hVDR. Three residues, previously identified by the hVDR docking model as key to bile acid activation - Arg-269 (R269; charge clamp to carboxylic acid group on bile acid side-chain), His-392 (H392; hydrogen bond to 3α-hydroxy group of LCA), Phe-417 (F417; stabilization of helix 12) - were mutated individually to two other amino acid resides. This produced a total of six-directed mutants: R269A, R269E, H392A, H392Y, F417A, and F417D.

We also made site-directed mutations at amino acid positions that showed differences between VDRs that are bile acid-sensitive (human, mouse, chicken, green-spotted pufferfish) and bile acid-insensitive (African clawed frog, sea lamprey, zebrafish). These mVDR mutations changed the amino acid residue(s) in mVDR to the corresponding residue(s) found in the bile-salt insensitive VDRs: Q286E (to corresponding residue found in xlVDR), S293 D (xlVDR), S293G (sea lamprey VDR), N319 D (lamprey VDR), N319K (zfVDR), and RCR363-365LCK (xlVDR), RCR363-365RIQ (zfVDR), RCR363-365ACR (lamprey VDR) (see Additional file 5). Lastly, we also tested the role of the H1-H3 'insertion' domain in mediating bile acid activation. Thus, we generated hVDR that lacked amino acid residues 165-218 (hVDR/Δins) and an hVDR construct where the insert domain was replaced with the insertion domain from the bile acid-insensitive xlVDR (hVDR/xlVDRins). The analogous constructs were also created for xlVDR: one lacking the insertion domain (xlVDR/Δins) and the other with the insertion domain from hVDR (xlVDR/hVDRins).

The two mutations at Arg269 (R269A and R269E) reduced the apparent affinity of 1α,25-dihydroxyvitamin D_3 _by over two orders of magnitude and abolished activation by LCA and 3-oxo-LCA. Similar effects were produced by the two mutations at His392 (H392A and H392Y) which reduced the apparent affinity of 1α,25-dihydroxyvitamin D_3 _by approximately 10-fold and 100-fold, respectively, and abolished activation by LCA and 3-oxo-LCA. The two mutations at Phe417 (F417A and F417D) both reduced the apparent affinity of 1α,25-dihydroxyvitamin D_3 _by approximately two orders of magnitude while retaining activation by LCA and 3-oxo-LCA but not LCA acetate. Interestingly, unsubstituted 5α-cholanic acid activated the R269A, R269E, H392A, H392Y, F417A, and F417D mutants weakly.

In general, the mutations at Glu-286, Ser-293, Asn-319, and Arg/Cys/Arg (363-365) had little effect on activation by 1α,25-dihydroxyvitamin D_3 _or bile acids compared to wild-type mVDR. Similarly, the four constructs that involved either deletion (hVDR/Δins and xlVDR/Δins) or swapping of the H1-H3 insertion domain (hVDR/xlVDRins and xlVDR/hVDRins) had little effect on activation by 1α,25-dihydroxyvitamin D_3 _or bile acids compared to wild-type hVDR. Thus, the H1-H3 insertion domain does not appear to play a major role in selectivity for bile acids.

### Pharmacophore models for *Tetraodon *VDR, PXR, and FXR

**Figure 7:**
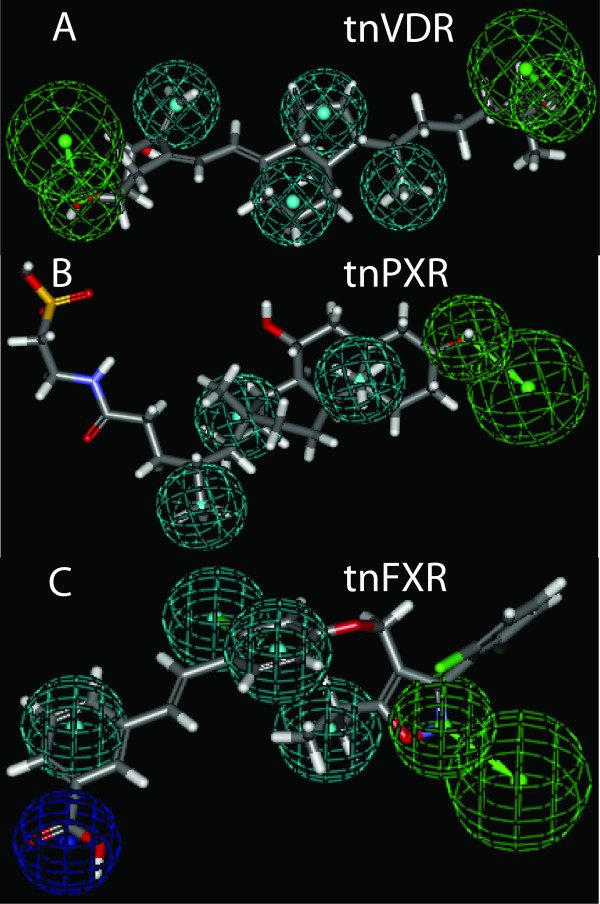
**Pharmacophore models of *Tetraodon *VDR, PXR, and FXR**. Pharmacophore models were developed for A) *Tetraodon *VDR (tnVDR), B) *Tetraodon *PXR (tnPXR), and C) *Tetraodon *FXR (tnFXR) using the HipHop method in Catalyst™. The structure-activity data used to develop the pharmacophore models are summarized in Additional File 6. A) The tnVDR pharmacophore contains 4 hydrophobes (cyan) and two hydrogen bond acceptors (green). 1,25-Dihydroxyvitamin D_3 _(calcitriol) was mapped to the pharmacophore. B) The tnPXR pharmacophore contains three hydrophobes (cyan) and one hydrogen bond acceptor (green). Taurochenodeoxycholic acid was mapped to the pharmacophore. C) The tnFXR pharmacophore contains four hydrophobes (cyan), one hydrogen bond acceptor (green), and one negative ionizable feature (blue). GW4064 was mapped to the pharmacophore.

Using the structure-activity data for tnVDR, tnPXR, and tnFXR (summarized in Additional File 6), we developed common features pharmacophore models for these three receptors (Figure 7). The tnVDR pharmacophore, mapped onto 1,25-dihydroxyvitamin D_3 _in Figure 7A, has four hydrophobes and two hydrogen bond acceptors. The tnPXR pharmacophore, mapped onto taurochenodeoxycholic acid in Figure 7B, shows three hydrophobes and one hydrogen bond acceptor, a smaller pharmacophore than that determined for hPXR [64]. The tnFXR pharmacophore, mapped onto GW4064 in Figure 7C, shows four hydrophobes, one hydrogen bond acceptor, and one negative ionizable feature.

### Homology modeling of *Tetraodon *PXR

**Figure 8:**
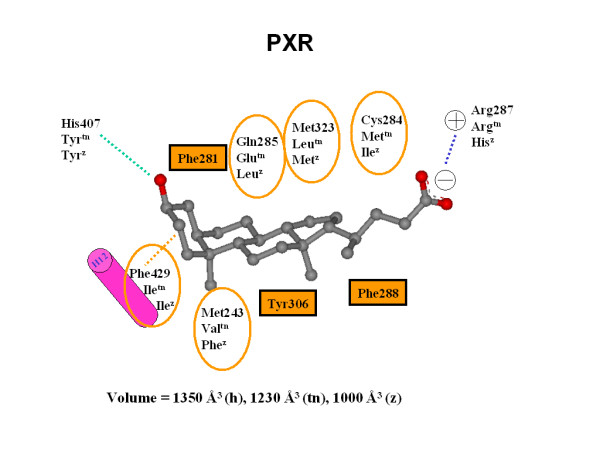
**PXR-ligand interactions**. Interaction map between the secondary bile acid lithocholic acid and PXRs. Key residues involved in binding are listed for human, *Tetraodon *(tn), and zebrafish (z) PXRs (the residue number is for human). Conserved resides throughout the three species are highlighted by black boxes. The hydrogen bond is shown by a green dashed line, the electrostatic charge interaction is presented as a blue dashed line, and the hydrophobic interaction is depicted as an orange dashed line. The ligand is shown as ball and stick presentation and colored according to atom types (gray = carbon, red-oxygen).

Structural studies of the LBD of hPXR reveal a large (~1,300 Å^3^), roughly spherical, hydrophobic LBP with the flexibility to accommodate large molecules such as hyperforin and rifampicin [30,32-34]. We can also see this by surface analysis of the co-crystallized ligands that cover a molecular weight range of 273-714 Da and a calculated ALogP (measure of hydrophobicity) range of 3.54-10.11 [65]. The homology model we generated of the LBD of tnPXR showed an LBP predicted to be slightly smaller (1,230 Å^3^) compared to hPXR (Figure 8), but larger than the estimated volumes of homology models of the LBPs of zebrafish PXR (~1,000 Å^3^) and *Xenopus laevis *PXRα (~860 Å^3^) that we reported previously [9,66]. The homology model results for tnPXR are consistent with the pharmacophore model for this receptor described above (Figure 7B).

**Figure 9:**
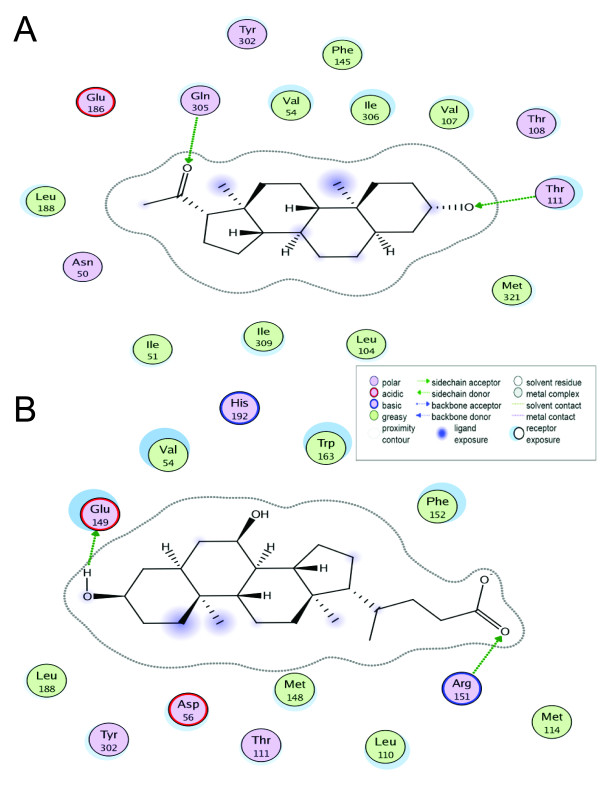
**Homology model of *Tetraodon *PXR**. Plot of interactions of ligands with tnPXR: A) the steroid 5β-pregnan-3,20-dione (pregnanedione) and B) the bile acid CDCA. The legend indicates the types of amino acid residues and amino acid-ligand interactions.

We performed molecular docking studies with two steroids (5β-pregnan-3,20-dione and 5α-androstan-3α-ol) and one bile acid (CDCA) that activated tnPXR in the luciferase assays (Figure 9). Docking studies show that the two steroids bind similar to the published crystallographic structure of 17β-estradiol bound to hPXR [35]. The carbonyl group of pregnanedione or hydroxyl group of androstanol at the C-3 position has a hydrogen bond with Thr-111, which corresponds to Ser-247 in hPXR. For pregnanedione, the carbonyl group at C-20 forms another hydrogen bond with Gln-305, as shown in Figure 6. Molecular docking indicated that the electrostatic interactions between CDCA and the conserved Arg-151 residue on helix 5 are important for binding to tnPXR. The hydroxyl group at the C-3 position forms a hydrogen bond with Glu-149, which is different from tnFXR, for which a His residue is involved in hydrogen bonding. For tnPXR, Tyr is in the position of this His residue, and adopts a different side-chain rotamer orientation to provide van der Waals contacts with the hydrocarbon scaffold of CDCA.

Homology modeling and docking studies of zebrafish PXR [9] show an important amino acid sequence difference (Met-243 in human PXR versus Phe at the corresponding position in zebrafish PXR). This residue is located at the bottom of the PXR LBP and has direct van der Waals contacts with A-ring and B-ring of the hydrocarbon scaffold of bile salts, specifically the methyl group at the C-10 position. In zebrafish PXR, the bulky and more rigid benzyl side-chain of the phenylalanine significantly narrow this portion of the zebrafish PXR LBP compared with human PXR which has the flexible side-chain of methionine-243, which may lead to preference for planar bile alcohols by zebrafish PXR. tnPXR has valine at the position corresponding to methionine-243 in human PXR, which allows enough room for either bent or planar formations of the A/B ring of bile salts.

### Homology modeling of *Tetraodon *FXR

For the homology model of tnFXR, molecular docking studies show that GW4064 adopts a similar orientation in the structural model as in the hFXR crystal structure [60]. The carboxyl group of GW4064 forms a strong electrostatic interaction with the conserved Arg residue in helix 5 of tnFXR. In addition, extensive hydrophobic contacts occur between the di-chloro-substituted benzyl ring of GW4064 and residues in helices 5, 7, 11 and 12 of tnFXR, which would stabilize the active conformation of receptor. CDCA also activated tnFXR in our experiments, but with weaker activity compared with its activity in hFXR. Sequence alignment indicated that most of the ligand contact residues identified in the crystallographic structure of hFXR [60] are conserved between tnFXR and hFXR. However, one important hydrogen bond interaction between the C-3 hydroxyl group of CDCA and hFXR is not present for tnFXR because Tyr-358 (hFXR) in helix 7 is substituted for by Phe in tnFXR.

**Figure 10:**
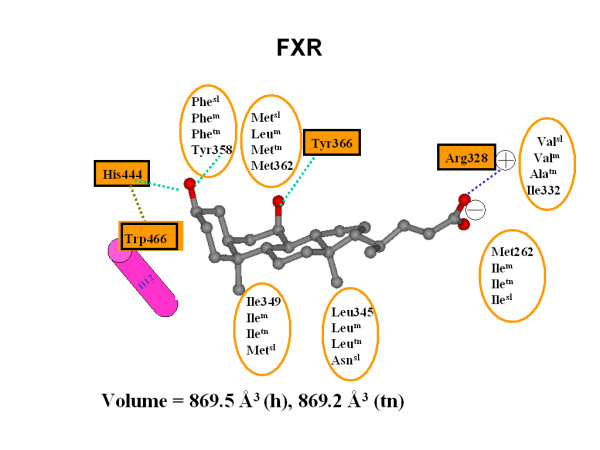
**FXR-ligand interactions**. Interaction map between the primary bile acid chenodeoxycholic acid and FXRs. Key residues involved in binding are listed for human, mouse (m), *Tetraodon *(tn), and sea lamprey (sl) FXRs (the residue number is for human). Conserved resides throughout the four species are highlighted by black boxes. Hydrogen bonds are shown by green dashed lines, the electrostatic charge interaction is presented as a blue dashed line, and the important cation-π interaction for FXR is depicted as a brown dashed line. The ligand is shown as ball and stick presentation and colored according to atom types (gray = carbon, red-oxygen).

There is an opening to solvent for the FXR LBP as seen in crystallographic structures of human or rat FXRs [59,60] and our homology models (Figure 10). The width of this opening is mostly controlled by two residues - Met-262 and Ile-332 in human FXR. For tnFXR, the residue corresponding to hFXR Ile-332 is alanine. Our model suggests that the LBP of tnFXR has a wider opening to solvent than hFXR, which may explain how tnFXR and not hFXR can accommodate cyprinol 27-sulfate, a C_27 _bile acid that has a longer side-chain and more hydroxyl groups than CDCA. The pharmacophore model for tnFXR summarized in Figure 7C is consistent with the homology modelling and docking results, in having multiple hydrophobic interactions, a hydrogen bond acceptor, and negative ionizable features for the bound ligand.

## Discussion

In this study, we characterize the FXR, VDR, and PXR of the green-spotted pufferfish *Tetraodon nigriviridis*, an actinopterygian fish that synthesizes mainly 5β-bile acids. This bile salt profile is similar to humans and most mammals, as well as many other actinopterygian fish, some reptiles, and some birds [13,23]. We find that the FXR and PXR of *Tetraodon *(tnFXR and tnPXR) are activated by common bent-shaped 5β-bile acids such as CDCA and less well by planar 5α bile alcohols, matching the endogenous primary bile salt profile of this species.

Our experimental and molecular modelling studies of tnFXR reveal a receptor that differs in multiple respects from sea lamprey and zebrafish FXRs [9] but is instead more similar to medaka FXRα [58] and mammalian FXRs. Homology modelling of tnFXR predicts an LBP with a wide opening to solvent (wider than crystallographic structure of hFXR [60]) and an overall architecture that differs markedly from homology models of sea lamprey and zebrafish FXRs, which are both predicted to have narrow LBPs that can accommodate planar bile alcohols but cannot accommodate bent bile acids such as CDCA. Docking studies of lamprey and zebrafish FXRs predict good binding to planar 5α-bile alcohols but not to bent 5β-bile acids, a prediction that is consistent with reporter assay experiments [9]. For tnFXR, docking studies show that the FXR-selective agonist GW4064 and bile acid CDCA adopt orientations in the LBP similar to those observed in the crystallographic structures of GW4064 bound to hFXR [60] and rat FXR bound to an analog of CDCA, respectively [59], lending further support to the conclusion that tnFXR has an overall LBP structure more similar to human and rat FXRs than to sea lamprey or zebrafish FXRs.

*Tetraodon *FXR has similar ligand selectivity to medaka FXRα (to which it shares high sequence identity in the LBD), including strong activation by GW4064 and the primary bile acid CDCA (both unconjugated and taurine-conjugated) [58]. Given that the activation of medaka FXRα upregulates the transcription of genes important in bile salt synthesis and transport, including the genes for CYP7A1 and bile salt export protein as well as the NHR repressor small heterodimer partner (SHP, NR0B2) [67], *Tetraodon *FXR seems likely to also be involved in regulation of bile salt biology. *Tetraodon *FXR and medaka FXRα have a substantially different ligand selectivity from sea lamprey FXR, which is not activated by GW4064, CDCA (or other 5β-bile acids), or T-0901317 [9]. This is not surprising because the primary bile salts of the sea lamprey (planar 5α-bile alcohols) are quite different from those of *Tetraodon *(CDCA, CA) and medaka (CDCA, CA, and C_27 _bile acids) [23,57,58]. The FXRs of *Tetraodon *and medaka also differ significantly from an FXR cloned and characterized from the little skate (*Leucoraja erinacea*, a cartilaginous fish), which was found to be insensitive to bile salts, even the 5β-bile alcohols (e.g., 5β-scymnol sulfate) produced by the little skate and many other cartilaginous fish [68]. However, the skate FXR showed significant differences in sequence from other vertebrate FXRs, including novel insertions, and there is the possibility that this receptor is actually orthologous to mammalian FXRβ (NR1H5), which are activated not by bile salts but instead by other steroidal compounds such as lanosterol [40].

Homology modelling of tnPXR predicts a receptor with an LBP with a volume (1,230 Å^3^) significantly larger than the estimated volumes of the LBPs of zebrafish PXR (~1,000 Å^3^) and *Xenopus laevis *PXRα (~860 Å^3^) [9,66] but smaller than the LBP of hPXR in crystallographic structures [30,32-34]. The homology model and docking studies of tnPXR are consistent with our studies of recombinant tnPXR in luciferase reporter assays that show activation by a wider range of ligands than can activate either zebrafish or *Xenopus *PXRs. For example, tnPXR is activated by a broad range of 5β- and 5α-bile salts and steroids similar to hPXR. In contrast, studies of recombinant zebrafish PXR show activation by a narrow range of planar 5α-bile alcohols [4,6,7]. The pharmacophore model for tnPXR is similar to models of hPXR [64,69,70] which have four hydrophobic features along with a hydrogen bond acceptor, although the tnPXR pharmacophore had three hydrophobic features and a hydrogen bond donor, consistent with a somewhat more restricted LBP limited to smaller ligands than hPXR [29,64]. Like hPXR, tnPXR has a pharmacophore model markedly different from that of chicken and zebrafish PXRs [29].

Overall, the ligand selectivity of tnFXR and tnPXR contrasts with that of sea lamprey FXR, zebrafish FXR, and zebrafish PXR, which are activated better by 5α bile alcohols, which are the main bile salts of jawless fish (Agnatha, which includes extant lampreys and hagfish) and Cypriniformes (which includes zebrafish) [9,23]. Unlike lampreys and zebrafish, *Tetraodon nigriviridis *has a bile salt profile of mainly bent 5β-bile acids such as CDCA, and would thus be predicted to have bile-salt-regulating NHRs that can recognize *Tetraodon *endogenous bile acids and not the 'ancestral' bile alcohols that comprise only a trace fraction of the *Tetraodon *bile salt pool. These results lend further significant support to the hypothesis that structural changes to the LBD of FXR and PXR throughout evolution have paralleled cross-species changes in bile salt profile [6,7,10,11,66]. In Additional file 7 bile salt variation and NHR bile salt sensitivity are overlaid on a fish phylogeny. So far, only a limited number of fish species have been characterized with respect to their FXRs, VDRs, and/or PXRs. As discussed in Additional file 7 a number of fish groups would be of particular interest for future studies of NHRs, including the Elasmobranchii (sharks, skates, rays), Chimaeriformes (chimaerae), Myxiniformes (hagfish), and lobe-finned fish (lungfish and coelacanths).

Our present and previous studies [6,7,9,10] with VDRs from various non-mammalian species have revealed some VDRs that are insensitive to bile acids (sea lamprey, African clawed frog) and others that are activated by secondary bile acids such as LCA (human, mouse, chicken, medaka, *Tetraodon*). In humans and some other mammals, enzymes from anaerobic bacteria in the caecum deconjugate and dehydroxylate primary bile acids such as CDCA, leading to secondary bile acids such as LCA that can produce toxicity to the intestinal and hepatobiliary tracts [15,21]. We and others have speculated that the ability of VDRs to bind secondary bile acids was acquired during vertebrate evolution as a protective mechanism against the potential toxicity of poorly water-soluble secondary bile acids such as LCA [6,7,51,52]. One challenge to this hypothesis is that there has been little study to date of the secondary bile salts (and the anaerobic bacterial intestinal flora that could generate such bile salts) of non-mammalian species. For example, it is not known whether *Tetraodon *or other actinopterygian fish species have physiologically important amounts of secondary bile acids in the intestinal tract. With these caveats in mind, we summarize our studies of VDRs across different species.

The structure-activity and molecular modelling data are both consistent with a tight, hydrophobic binding pocket for bile acids in the human VDR LBP that can bind bile acids with an oxo or single hydroxyl group at the C-3 position but not bile acids with substituents at the C-7 or C-12 positions (including unnatural bile acids with a single hydroxyl group on C-7 or C-12 and no substituent on C-3). Molecular modeling predicts an hVDR bile acid binding pocket that is predominantly hydrophobic but with polar features that permit hydrogen bond interaction with the 3α-hydroxy or 3-oxo group on the A ring of the bile acid and electrostatic interactions with the bile acid side-chain as described above. Bile acids that have hydroxyl groups on steroid rings B and/or C (CDCA, DCA, CA, 7α-hydroxy-5β-cholanic acid, 7β-hydroxy-5β-cholanic acid, and 12α-hydroxy-5β-cholanic acid) do not interact favourably with the more hydrophobic and sterically constrained portion of the hVDR bile acid binding pocket, consistent with the lack of activity of these bile acids in transactivation assays. On the contrary, the unsubstituted and hydrophobic B, C, and D rings of LCA complement well the lipophilic portion of hVDR pocket and can form numerous van der Waals interactions. We suggested that the unique physicochemical arrangement of the hVDR ligand binding cavity provides the structural basis for selective activation by LCA and its derivatives [9].

It is less clear, even with our mutagenesis studies, exactly what changes mediate the cross-species differences in VDR pharmacology. Our studies rule out the helix 1 - helix 3 'insertion' domain as playing a role in the cross-species differences in activation by secondary bile acids. This insert is disordered in human and mouse VDRs and shows highly variable sequences and lengths across species [71-74]. Our site-directed mutagenesis experiments do confirm the previous prediction that Arg-274 and His-397 play a key role in interacting with bile acids [50,51]. Interestingly, Arg-274 and His-397 are conserved in the bile salt-insensitive xlVDR, indicating that other determinants underlie the differences in bile salt pharmacology between xlVDR and bile-salt responsive VDRs.

## Conclusions

Our studies provide further important evidence of the relationship between FXR, PXR, and VDR ligand selectivity and cross-species variation in bile salt profiles. Zebrafish and the green-spotted pufferfish *Tetraodon nigriviridis *provide a clear contrast in having markedly different primary bile salt profiles (planar bile alcohols for zebrafish and sterically bent bile acids for the pufferfish) and correspondingly contrasting receptor selectivity that matches the differences in endogenous ligands. In contrast, *Tetraodon *has a bile salt profile and receptor ligand selectivity similar to humans. Our observations present an integrated picture of co-evolution of bile salt structure and the binding pockets of three nuclear hormone receptors.

## Methods

### Bile sample analysis

Bile salts from the green-spotted pufferfish were analyzed by HPLC and ESI/MS/MS using methods previously described [23,75].

### Chemicals

The sources of chemicals were as follows: GW4064 (Sigma, St. Louis, MO, USA); T-0901317 (Axxora, San Diego, CA, USA); 3α,7α,12α,24-tetrahydroxy-5α-cholan-24-sulfate (5α-petromyzonol sulfate; Toronto Research Chemical, Inc., North York, ON, Canada); 1α,25-(OH)_2_-vitamin D_3_, Nuclear Receptor Ligand Library (76 compounds known as ligands of various NHRs; BIOMOL International, Plymouth Meeting, PA, USA). All other commercially purchased steroids and bile salts were obtained from Steraloids (Newport, RI, USA). Norlithocholic acid, 3β-hydroxy-5α-cholan-24-oic acid, 3β-hydroxy-5β-cholan-24-oic acid, 7α-hydroxy-5β-cholan-24-oic acid, 7β-hydroxy-5β-cholan-24-oic acid, and 12α-hydroxy-5β-cholan-24-oic acid were generously donated by the laboratory of A.F. Hofmann (University of California - San Diego, La Jolla, CA, USA).

### Cloning and expression of full-length VDRs

Full-length VDRα sequences from medaka (*Oryzias latipes*), zebrafish, and green spotted pufferfish were identified within each species genome by conducting a generalized BLAST search using a query sequence representing the P box domain of human VDR located within the highly conserved DNA binding region for the NHR superfamily. Full-length transcripts were subsequently determined by identifying transcriptional start and stop codons for each gene. cDNAs containing a complete open reading frame for each gene were produced by PCR using primer sets that spanned the entire nucleic acid sequence for each gene including start and stop codons. cDNAs were produced from extracts of fish liver total RNA. Livers were homogenized with 1 mL RNA Bee (Tel-Test, Inc. Friendswood, TX, USA) using a stainless steel Polytron homogenizer (Kinematica, Lucerne, Switzerland) followed by cleanup and on- column DNase treatment using an RNeasy Mini Kit (Qiagen, Valencia, CA, USA).

RNA was eluted with 30 μl RNase-free water. RNA quantity and quality were verified using an Agilent 2100 Bioanalyzer (Agilent Technologies, Santa Clara, CA, USA) and NanoDrop^® ^ND-1000 spectrophotometer (ThermoScientific, Wilmington, DE, USA). First strand cDNA was made from total RNA (1-3 μg) and diluted with RNase-free water to a final volume of 10 μl, and 1 μl oligo(dT)_15 _(500 μg/ml; Promega, Madison, WI, USA) and 1 μl 10 mM dNTPs were mixed with diluted RNA to yield a final volume of 20 μl. The mix was heated to 65°C for 5 min and chilled on ice for 2 min. Following centrifugation, 4 μl 5X first-strand buffer (Invitrogen, Carlsbad, CA, USA), 2 μl of 0.1 M DDT, and 1 μl RNase OUT Inhibitor (40 U/μl; Invitrogen) were added to each reaction and heated to 37°C. Following a 2 min incubation, 1 μl Superscript Reverse Transcriptase (200 U/μl; Invitrogen) was added to each reaction and mRNA reverse transcribed at 37°C for 1 h. All reverse transcription (RT) reactions were then inactivated by incubating at 70°C for 15 min. cDNAs were stored at -20°C until PCR. PCR primers for teleost VDRα were designed using PrimerQuest (Integrated DNA Technologies, Coralville, IA, USA). PCR primers were flanked by restriction sites for incorporation and transfer between appropriate cloning and expression vectors. For each 25-μl PCR reaction, first-strand cDNAs were amplified using 2 μl (100-300 ng) first-strand cDNA, 9 μl RNase-free water, 0.75 μl 10 μM forward primer (0.3 μM), 0.75 μl 10 μM reverse primer (0.3 μM), and 12.5 μl 2X Advantage Taq PCR Master Mix (Clontech, Mountain View, CA, USA). PCR reaction conditions were: 95°C for 1.5 min followed by 35 cycles of 94°C for 15 s, 55°C for 30 s, and 72°C for 1 min. PCR products for each teleost VDRα sequence were cloned into the TA cloning vector pCR2.1 (Invitrogen, Carlsbad, CA) as per manufacturer's suggestions. VDR's were subsequently excised from pCR 2.1 using *Eco*RI and *Bam*HI and inserted unidirectionally into the expression vector pSG5. Proper orientation of VDRα's within the vector was confirmed by PCR screening and sequencing in both directions.

HepG2 cells were cultured in T75 flasks with vented caps (Corning, Corning, NY) using MEM containing head-inactivated fetal bovine serum (10%), 1X sodium pyruvate, 1X nonessential amino acids, and 1% penicillin/streptomycin. Cells were maintained at 37°C with 5% CO_2 _and split regularly at 70-80% confluency. For transient transfection, HepG2 cells were seeded at a density of 1 × 10^5 ^cells per well in 24 well plates in antibiotic-free MEM. Wells were transfected over night with either empty pSG5 vector (control), medaka VDRα, zebrafish VDRα, or *Tetraodon *VDRα. Each well contained 299 ng pSG5-VDRα for either (medaka, zebrafish, or *Tetraodon*), 64 ng hCYP3A4-Luc reporter construct (XREM), and 15 ng of the *Renilla *normalizing plasmid (pRL-CMV). Luciferase reporter assay experiments were performed as previously described [58].

Plasmids containing human organic anion transporting polypeptide (Oatp1a1; Slco1a1), as well as the reporter construct tk-UAS-Luc and the 'empty' vector PM2, were generously provided by SA Kliewer, JT Moore, and LB Moore (GlaxoSmithKline, Research Triangle Park, NC, USA). To permit comparison between species and to avoid mismatching of non-mammalian receptors with mammalian retinoid X receptor, co-factors, and chromatin remodeling factors, all receptors were studied as LBD/GAL4 chimeras. For the GAL4/LBD expression constructs, the reporter plasmid is tk-UAS-Luc, which contains GAL4 DNA binding elements driving luciferase expression. The cloning of LBDs from hFXR, hVDR, mouse VDR (mVDR), and zebrafish VDR has been previously reported [6,7,9].

The LBD of chicken VDR was cloned from RNA extracted from the LMH cell line. The LBD of *Xenopus laevis *VDR was cloned from RNA extracted from the A6 cell line. The LBDs of tnFXR, tnVDR, and tnPXR were cloned from total RNA extracted from *Tetraodon *liver using sequence information from the draft *Tetraodon nigriviridis *genome [76]. The LBDs of chVDR (amino acid residues 113-451), xlVDR (amino acid residues 91-422), tnFXR (amino acid residues 180-463), tnVDR (amino acid residues 91-426), and tnPXR (amino acid residues 202-483) were inserted into the pM2-GAL4 vector to create GAL4/LBD chimeras.

Site-directed mutations of mVDR were performed using the QuikChange II mutagenesis kit (Stratagene, La Jolla, CA, USA). All mutations were confirmed by sequencing of both DNA strands. To explore the importance of the H1-H3 insert in ligand selectivity, four constructs were created. Thus, we generated hVDR/Δins that lacked amino acid residues 165-218 (hVDR/Δins) and a hVDR construct where the insert domain was replaced with the insertion domain from bile acid-insensitive xlVDR (hVDR/xlVDRins; hVDR residues 158-223 replaced by xlVDR residues 160-218). The analogous constructs were also created for xlVDR: one lacking the insertion domain (xlVDR/Δins; missing amino acid residues 166-211) and the other (xlVDR/hVDRins; xlVDR residues 160-218 replaced by hVDR residues 158-223). The chimeras were generated by synthesis of double-stranded DNA (Genscript, Piscataway, NJ) which was inserted into the pM2-GAL4 vector.

### Cell lines

The creation of a HepG2 (human liver) cell line stably expressing the human Na^+^-taurocholate cotransporter (NTCP; SLC10A1) has been previously reported [7]. HepG2-NTCP cells were grown in modified Eagle's medium-α containing 10% fetal bovine serum and 1% penicillin/streptomycin. The cells were grown at 37°C in 5% CO_2_. The chicken LMH hepatoma cell line (ATCC, Manassus, VA, USA) was grown in Waymouth's MB 752/1 medium (ATCC) with 10 fetal bovine serum. The *Xenopus laevis *A6 kidney cell line (ATCC) was grown in 75% NCTC 109 medium, 15% distilled water, and 10% fetal bovine serum at 26°C in 2% CO_2_. Except as noted above, all media and media supplements for the HepG2 and A6 cell lines were obtained from Invitrogen (Carlsbad, CA, USA). Co-transfections and transactivations assays were performed as previously described [77]. The maximal activators and their concentrations were as follows: chVDR, xlVDR, and tnVDR - 200 nM 1α,25-(OH)_2_-vitamin D_3_; tnFXR - 5 μM GW4064; tnPXR - 50 μM 5α-androstan-3α-ol. All comparisons to maximal activators were done within the same microplate. Luciferase data were normalized to the internal β-galactosidase control and represent means ± SD of the assays.

### Common features pharmacophore development and database screening

Computational molecular modeling studies were carried out using Catalyst™in Discovery Studio 2.5.5 (Accelrys, San Diego, CA, USA) by methods previously described [29]. Common features pharmacophores were developed based on the compounds tested against tnFXR, tnPXR and tnVDR using the HipHop method [78,79]. A HipHop pharmacophore attempts to describe the arrangement of key features that are important for biological activity.

### Molecular modeling studies

A structural model of the LBDs of the tnFXR and tnPXR were constructed using the Molecular Operating Environment (MOE; Chemical Computating Group, Montreal, Canada) Homology Model module. The modeling template used for FXR is the published crystal structure of rat FXR in complex with 6α-ethyl-chenodeoxycholic acid (PDB ID = 1OSV) [59] (this structure was chosen because it has bound bile acid unlike available crystal structures of hFXR). The template for PXR is hPXR co-crystallized with SR12813 (PDB ID = 1nrl) [33]. Several energy minimization-based refinement procedures were implemented on the initial model, and the quality of the final model was confirmed by the WHATIF-Check program. Estimation of the volume of the LBP for the crystallographic structures and homology models described above were determined using CASTp http://sts.bioengr.uic.edu/castp/calculation.php[80].

Molecular docking studies were performed by the GOLD docking program [81]. In addition to the docking studies with tnPXR and tnFXR, LCA was docked into the crystal structure for human VDR (PDB accession number 1DB1). During the docking process, the protein was held fixed while full conformational flexibility was allowed for ligands. For each ligand, 30 independent docking runs were performed to achieve the consensus orientation in the LBP.

## Authors' contributions

NA and SE performed molecular modelling studies, including those that provided the basis for the site-directed mutagenesis experiments. LRH performed the analysis of bile salts of the green-spotted pufferfish. EMK and SWK performed the cloning, expression, and analysis of full-length VDRs in three actinopterygian fish species. EJR and MDK cloned *Tetraodon *FXR, *Tetraodon *PXR, chicken VDR, *Xenopus laevis *VDR, and *Tetraodon *VDR, as well as all site-directed mutants of mouse VDR. MDK performed the function assays of all GAL4/LBD chimeric receptors and drafted the manuscript. All authors contributed to, read, and approved the final manuscript.

## Supplementary Material

Additional file 1 **Analyses of biliary bile salts from the green-spotted pufferfish**. Click here for file

Additional file 2 **Activation data for *Tetraodon *FXR and PXR by bile salts, steroids, and synthetic ligands**. Click here for file

Additional file 3 **Activation data for VDRs, including the site-directed mutants of mouse VDR**. Click here for file

Additional file 4 **The ligand binding pocket of human VDR**. Surface representation of human VDR ligand binding pocket. Click here for file

Additional file 5 **Amino acid sequence alignment for VDRs (including the four chimeric constructs involving the H1-H3 insertion domain) and human PXR**. Click here for file

Additional file 6 **Data for pharmacophore modeling of *Tetraodon *nuclear hormone receptors**. Click here for file

Additional file 7 **Fish phylogeny, bile salt variation, and nuclear hormone receptor ligand specificity**. Click here for file
