# Supplementary material for: The evolution of farnesoid X, vitamin D, and pregnane X receptors: insights from the green-spotted pufferfish (Tetraodon nigriviridis) and other non-mammalian species
Source: BMC Biochem. 2011 Feb 3;12:5. doi: 10.1186/1471-2091-12-5 (PMC3042382; doi:10.1186/1471-2091-12-5)
Supplement: Additional file 1 — Analyses of biliary bile salts from the green-spotted pufferfish. [file 1471-2091-12-5-S1.PDF]

ADDITIONAL FILE 1

The evolution of farnesoid X, vitamin D, and pregnane X receptors: insights from the green-spotted pufferfish (*Tetraodon nigriviridis*) and other non-mammalian species

Matthew D Krasowski, Ni Ai, Lee R Hagey, Seth W. Kullman, Erica J Reschly, Sean Ekins

Supplementary Figure S1

Analyses of biliary bile from green-spotted pufferfish

Electrospray ionization-tandem mass spectrometry (ESI/MS/MS) spectra are annotated with probable matches based on *m/z* ratios and corroborations with other analyses including high-performance liquid chromatography (HPLC), gas chromatography/mass spectrometry, and thin-layer chromatography. HPLC traces are annotated with matches based on retention time in comparison with reference standards.

A) In this HPLC trace, the major bile acids of the green-spotted pufferfish are taurocholic acid and taurochenodeoxycholic acid.

B) In this ESI/MS/MS analysis focused on broad identification of bile salts in the green-spotted pufferfish, the major peaks have *m/z* ratios consistent with the ions of taurocholic acid (peak A) and taurochenodeoxcholic acid (peak B). Additional adducts of taurocholic acid and taurochenodeoxycholic acid are also identified (peaks D-G). A small peak with an *m/z* ratio consistent with the pentahydroxy C_{27} bile alcohol sulfate is also identified.

C) In this ESI/MS/MS analysis focused on parents of sulfates, the minor populations of C_{27} and C_{29} bile alcohols in the green-spotted pufferfish are shown.

Additional file 1
Supplemental Fig. S1A

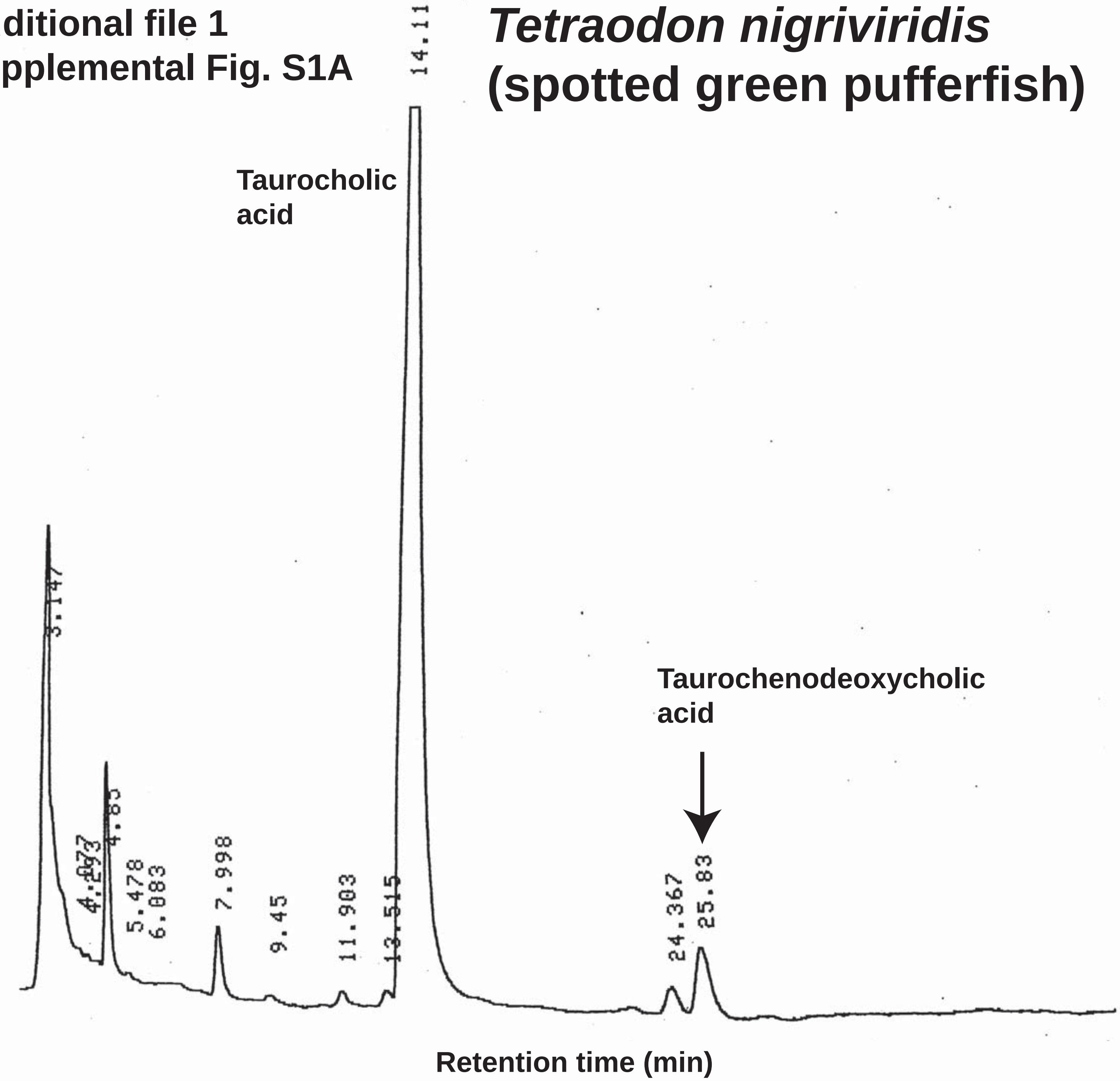

Additional file 1
Supplemental Fig. S1B

Tetraodon nigriviridis (spotted green pufferfish)

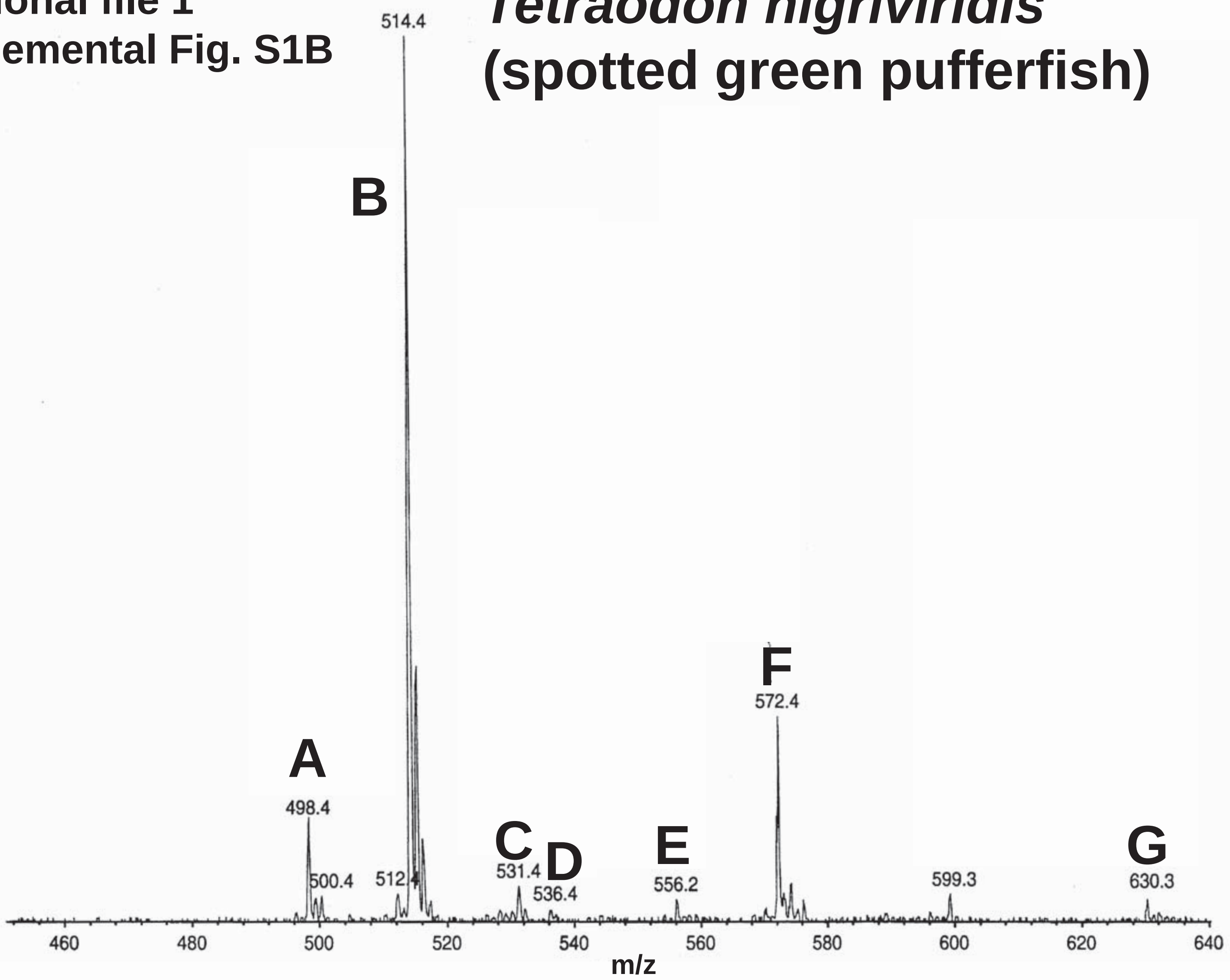

	Class	# of Hydroxyls	Conjugation	Adducts (if any)
A	C_{24} acid	2	Taurine	
B	C_{24} acid	3	Taurine	
C	C_{27} alcohol	5	Sulfate	
D	C_{24} acid	3	Taurine	Na
E	C_{24} acid	2	Taurine	NaCl
F	C_{24} acid	3	Taurine	NaCl
G	C_{24} acid	3	Taurine	2NaCl

Additional file 1
Supplemental Fig. S1C

Tetraodon nigriviridis (spotted green pufferfish)

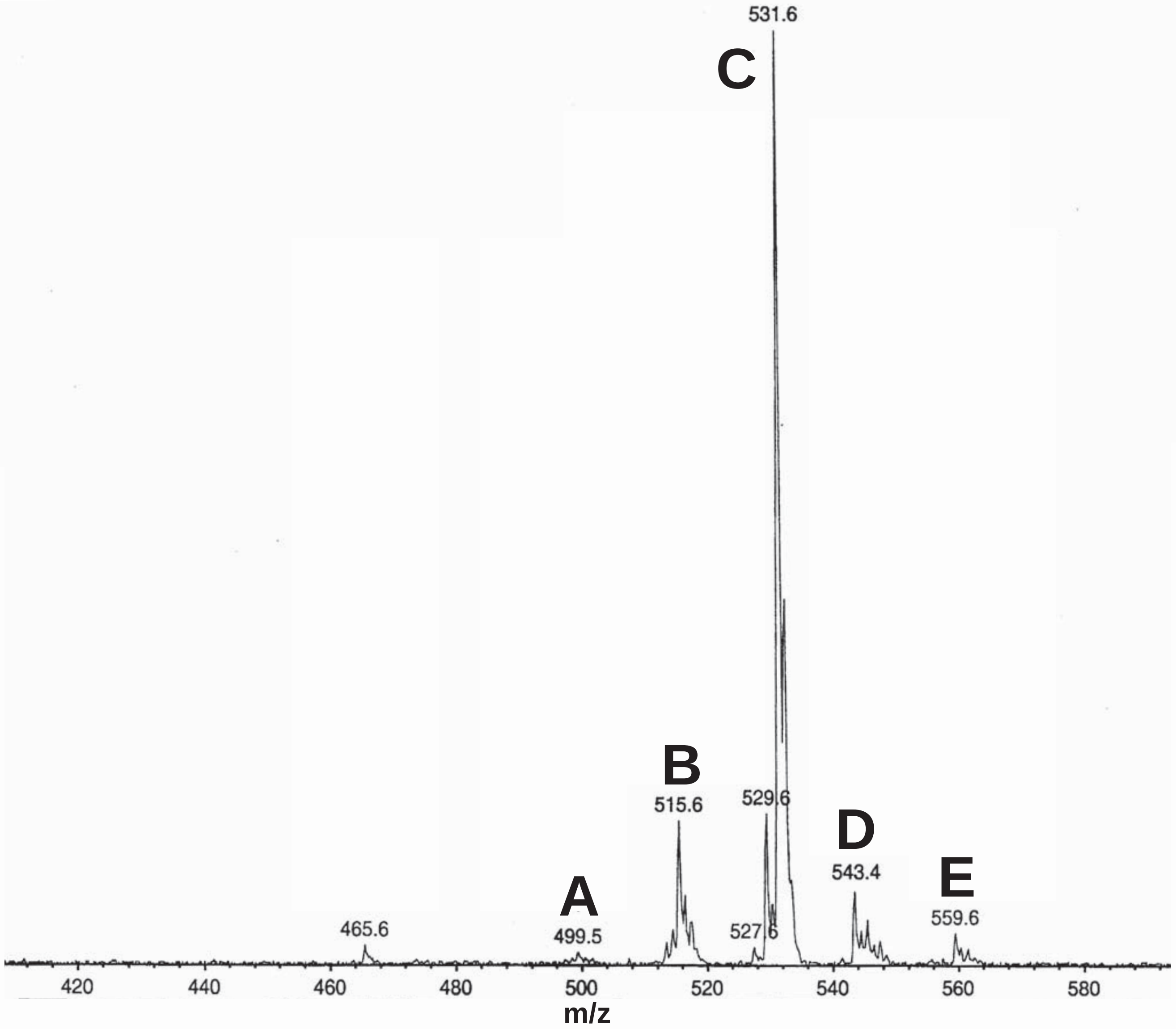

	Class	# of Hydroxyls	Conjugation
A	C_{27} alcohol	3	Sulfate
B	C_{27} alcohol	4	Sulfate
C	C_{27} alcohol	5	Sulfate
D	C_{29} alcohol	4	Sulfate
E	C_{29} alcohol	5	Sulfate
