# Supplementary material for: The evolution of farnesoid X, vitamin D, and pregnane X receptors: insights from the green-spotted pufferfish (Tetraodon nigriviridis) and other non-mammalian species
Source: BMC Biochem. 2011 Feb 3;12:5. doi: 10.1186/1471-2091-12-5 (PMC3042382; doi:10.1186/1471-2091-12-5)

Additional file 4 – The ligand binding pocket of human VDR

Surface representation of human VDR ligand binding pocket. The surface is colored by atom type (nitrogen – blue; oxygen – red; sulphur – yellow; carbon – white; hydrogen – green). Residues inside the pocket are also displayed.

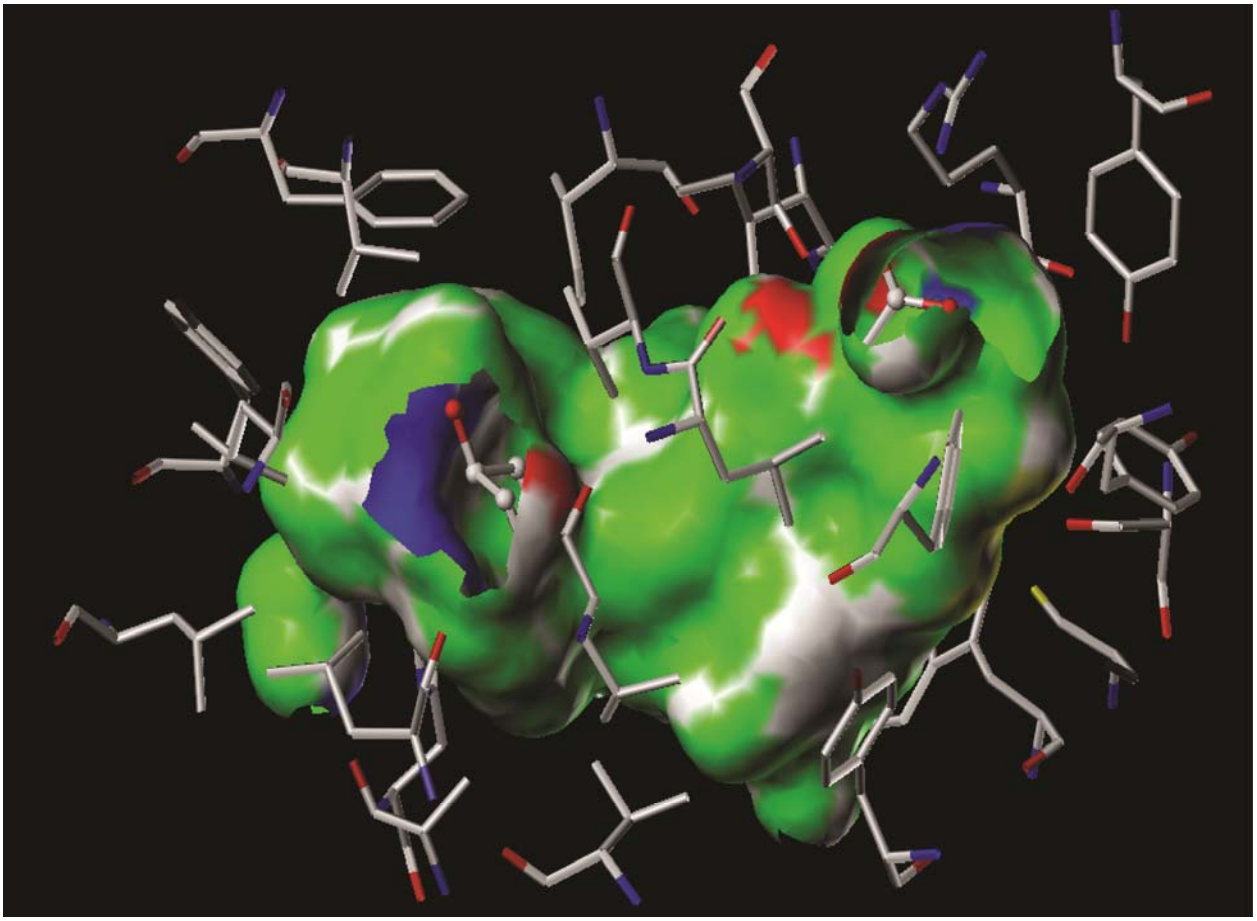

Supplement: Additional file 4 — The ligand binding pocket of human VDR. Surface representation of human VDR ligand binding pocket. [file 1471-2091-12-5-S4.PDF]
