# Supplementary material for: The evolution of farnesoid X, vitamin D, and pregnane X receptors: insights from the green-spotted pufferfish (Tetraodon nigriviridis) and other non-mammalian species
Source: BMC Biochem. 2011 Feb 3;12:5. doi: 10.1186/1471-2091-12-5 (PMC3042382; doi:10.1186/1471-2091-12-5)
Supplement: Additional file 5 — Amino acid sequence alignment for VDRs (including the four chimeric constructs involving the H1-H3 insertion domain) and human PXR. [file 1471-2091-12-5-S5.PDF]

Additional file 5

Amino acid sequence alignment of VDRs and human PXR

```
VDR_hum_noins = human VDR without H1-H3 insert

VDR_hum_xlins = human VDR with H1-H3 insert replaced by that from Xenopus laevis
                  VDR

VDR_xl_noins = Xenopus laevis VDR without H1-H3 insert

VDR_xl_humins = Xenopus laevis VDR with H1-H3 insert replaced by that from human
                  VDR

                       __Helix-1________
VDR_human         122 PKLSEEQQRIIAILLDAHHKTYDPTYSDFCQFRPPVRVNDGGGSHPSRPNSRHTPSF-SG
VDR_hum_noins         PKLSEEQQRIIAILLDAHHKTYDPTYSDFCQFRPPVRVNDGGG-----------------
VDR_hum_xlins         PKLSEEQQRIIAILLDAHHKTYDPTYSDFCQFRPPVRENVDPFRRITRSSSVHTQGSPSE
VDR_dog               PKLSEEQQRIIAILLDAHHKTYDPTYADFTQFRPPVRGDGGGGSHSSRPSSAHTPSV-SG
VDR_mouse             PKLSEEQQHIIAILLDAHHKTYDPTYADFRDFRPPIRADVSTGSYSPRP----TLSF-SG
VDR_chicken           PKLSEEQQKVIDTLLEAHHKTFDTTYSDFNKFRPPVRSKFSSRMATHSSSVVSQDFS-SE
VDR_xen_laevis        PKISDEQQKMIDILLEAHRKTFDTTYSDFNKFRPPVRENVDPFRRITRSSSVHTQGSPSE
VDR_xl_noins          PKISDEQQKMIDILLEAHRKTFDTTYSDFNKFRPPVRENVDPF-----------------
VDR_xl_humins         PKISDEQQKMIDILLEAHRKTFDTTYSDFNKFRPPVRVNDGGGSHPSRPNSRHTPSF-SG
VDR_salmon            PRLNDEQSQVIASLVEAHHKTYDDSYSDFSRFRPPVREGPVTRSASRAASLHSLSDA-SS
VDR_stickleback       PRLNEEQSRIISSLVEAHHKTFDASYSDFSRFRPPVREGPVTRSASRAASLHSLSDA-SS
VDR_Tetraodon         PRLNEEQARIISSLVEAHHKTYDTSYSDFSRFRPPVRERPVTRSASRAASLHSLSDA-SS
VDR_zebrafish         PRLSDEQMQIINSLVEAHHKTYDDSYSDFVRFRPPVREGPVTRSASRAASLHSLSDA-SS
VDR_medaka            PRLNEEQARIISSLVEAHHKTYDASYSDFSRFRPPVRDGPVTRSASRAASLHSLSDA-SS
VDR_lamprey           PQLLEEQERLIATLIEAHRKTYDASYSDFSQFRPPKRGDGSPECRNATNPFLMSLLN-SD
PXR_human         141 QGLTEEQRMMIRELMDAQMKTFDTTFSHFKNFRLPGVLSSGCELPESLQAP-SREEA-AK

                                                                    Helix-3_____
VDR_human         181 DSSSSCSDHCITSSDM-MDSSSFSNLDLSEEDSDDPSVTLELSQLSMLPHLADLVSYSIQ
VDR_hum_noins         ------------------------------------SVTLELSQLSMLPHLADLVSYSIQ
VDR_hum_xlins         DSDVFTSSPDSSEHGFFSASLFGQFEYSSMGGKSGELS--------MLPHIADLVSYSIQ
VDR_dog               DSSSSCSDHYPALLDV-MEPTSFSNLDLREEDSDDSSLTLDLSQLSMLPHLADLVSYSIQ
VDR_mouse             DSSSNS-DLYTPSLDM-MEPASFSTMDLNEEGSDDPSVTLDLSPLSMLPHLADLVSYSIQ
VDR_chicken           DSNDVFGSDAFAAFPEPMEPQMFSNLDLSEESDESPSMNIELPHLPMLPHLADLVSYSIQ
VDR_xen_laevis        DSDVFTSSPDSSEHGFFSASLFGQFEYSSMGGKSGELS--------MLPHIADLVSYSIQ
VDR_xl_noins          -----------------------------MGGKSGELS--------MLPHIADLVSYSIQ
VDR_xl_humins         DSSSSCSDHCITSSDM-MDSSSFSNLDLSEEDSDDPSVTLELSQLSMLPHIADLVSYSIQ
VDR_salmon            DSFNHSPESVDTKLMNFSSLLMMYQDSAGSPDSSEDD----GSKLSMLPHLADLVSYSIQ
VDR_stickleback       DSFNHSPESVDTKMNFTSLLMMYQDGASSPDSSEED------TKLSMLPHLADLVSYSIQ
VDR_Tetraodon         DSLNHSPESMDTKLNFSNLLMMYQDGVSSSDSSEEE------TKLSMLPHLADLVSYSIQ
VDR_zebrafish         DSFNHSPESVDTKLNFSNLLMMYQDSG-SPDSSEEDQQS----RLSMLPHLADLVSYSIQ
VDR_medaka            DSFNHSPESVDTKMNFSSLLMMYQDGVNSPDSSEEDT------KLSMLPHLADLVSYSIQ
VDR_lamprey           MD-------------------------ELPKASASGAEAAAGDELSMLPHLADLVSYSIQ
PXR_human         199 WSQVRKDLCSLKVSLQL-RGEDGSVWNYKPP-----ADSGGKEIFSLLPHMADMSTYMFK

                      Helix-3          Helix-4    Helix-5
VDR_human         240 KVIGFAKMIPGFRDLTSEDQIVLLKSSAIEVIMLRSNESFTMDD-MSWTCGNQDYKYRVSD
VDR_dog               KVIGFAKMIPGFRDLTSEDQIVLLKSSAIEVIMLRSNQSFIMDD-MSWTCGSQDYKYRVSD
VDR_mouse             KVIGFAKMIPGFRDLTSDDQIVLLKSSAIEVIMLRSNQSFTMDD-MSWDCGSQDYKYDITD
VDR_chicken           KVIGFAKMIPGFRDLTAEDQIALLKSSAIEVIMLRSNQSFTMED-MSWTCGSNDFKYKVSD
VDR_xen_laevis        KIIGFAKMIPGFRDLIAEDQIALLKSSVIEVIMLRSNQSFSLDD-MSWTCGSEDFKYKVDD
VDR_salmon            KVIGFAKMIPGFRGLTAEDQTALLKSSAIEIIMLRSNQSFNPED-MSWSCGGPDFKYCVND
```

```
VDR_stickleback     KVIGFAKMIPGFRELTAEDQIALLKSSAIEIIMLRSNQSFSLED-MSWSCGGPDFKYCIND
VDR_Tetraodon       KVIGFAKMIPGFRDLTAEDQIALLKSSAIEIIMLRSNQSFSLED-MSWSCGGPDFKYCITD
VDR_zebrafish       KVIGFAKMIPGFRDLTAEDQIALLKSSAIEIIMLRSNQSFSLED-MSWSCGGPDFKYCIND
VDR_medaka          KVIGFAKMIPGFRDLTAEDQIALLKSSAIEIIMLRSNQSFSLED-MSWSCGGPDFKYCVND
VDR_lamprey         KVIGFAKMIPGFKELCTEDQISLLKASAIEIIILRSNESFTMED-NSWTCGSNEFKYQIGD
PXR_human       253 GIISFAKVISYFRDLPIEDQISLLKGAAFELCQLRFNTVFNAET-GTWECGR--LSYCLED

                            Helix-7             Helix-8                ___Helix-9_
VDR_human       304 VTKAGHSLELIEPLIKFQVGLKKLNLHEEEHVLLMAICIVSPDRPGVQDAALIEAIQDRLS
VDR_dog             VAKAGHSLELIEPLIKFQVGLKKLNLHEEEHVLLMAICILSPDRPGVQDAALVEALQDRLS
VDR_mouse           VSRAGHTLELIEPLIKFQVGLKKLNLHEEEHVLLMAICIVSPDRPGVQDAKLVEAIQDRLS
VDR_chicken         VTQAGHSMDLLEPLVKFQVGLKKLNLHEEEHVLLMAICILSPDRPGVQDTSLVESIQDRLS
VDR_xen_laevis      VTQAGHNMELLEPLVKFQVGLKKLDLHEEEHVLLMAICILSPDRPGLQDKALVESIQDRLS
VDR_salmon          VTKAGHTLDLLEPLVKFQVGLKKLKLHEEEHVLFMAICLLSPDRPGVQDHAKIEVPQDRLS
VDR_stickleback     VTKAGHTLDLLEPLVKFQVGLKKLNLHEEEHVLLMAICLLSPDRPGVQDHGRVEQLQDHLS
VDR_Tetraodon       VTKAGHTLELLEPLVKFQVGLKKLNLHEEEHVLLMAICLLSPDRPGVQDHRHVEKLQDHLS
VDR_zebrafish       VTKAGHTLELLEPLVKFQVGLKKLKLHEEEHVLLMAICLLSPDRPGVQDHVRIEALQDRLC
VDR_medaka          VTKAGHTLELLEPLVKFQVGLKKLNLHEEEHVLLMAICLLSPDRPGVQDHARIEQLQDRLS
VDR_lamprey         VMQAGHKLELLEPLVKFQVNMKKLDLHEAEHVLLMAICLFSPDRPGVQDRCRVEEVQEHLT
PXR_human       311 T-AGGFQQLLLEPMLKFHYMLKKLQLHEEEYVLMQAISLFSPDRPGVLQHRVVDQLQEQFA

                     Helix-9__       ____Helix-10___________________
VDR_human       361 NTLQTYIRCRHPPPGSHLLYAKMIQKLADLRSLNEEHSKQYRCLSFQPECSMKLTPLVLEV
VDR_dog             NTLQTYIRCRHPPPGSHLLYAKMIQKLADLRSLNEEHSKQYRCLSFQPECSMKLTPLVLEV
VDR_mouse           NTLQTYIRCRHPPPGSHQLYAKMIQKLADLRSLNEEHSKQYRSLSFQPENSMKLTPLVLEV
VDR_chicken         DILQTYIRCRHPPPGSRLLYAKMIQKLADLRSLNEEHSKQYRCLSFQPEHSMQLTPLVLEV
VDR_xen_laevis      STLQTYILCKHPPPGSRLLYAKMIQKLADLRSLNEEHSKQYRSISFLPEHSMKLTPLMLEV
VDR_salmon          EVLQAYIRVNHP--GGRLLYARMIQKLADLRSLNEEHSKQYRSLSFQPEHSMQLTPLVLEV
VDR_stickleback     ETLQAYIQVNHP--GGRLLYAKMIQKLADLRSLNEEHSKQYRSLSFQPEHSMQLTPLVLEV
VDR_Tetraodon       EILQAYIRVNHP--GGRLLYAKMIQKLADLRSLNEEHSKQYRSLSFQPEHSMQLTPLVLEV
VDR_zebrafish       DVLQAYIRIQHP--GGRLLYAKMIQKLADLRSLNEEHSKQYRSLSFQPEHSMQLTPLVLEV
VDR_medaka          EALQAYIRVNHP--GGRLLYAKMIQKLADLRSLNEEHSKQYRSLSFQPEHSMQLTPLVLEV
VDR_lamprey         ETLRAYIACRHPLSCKHMLYTKMVEKLTELRSLNEEHSKQYLQISQDAVNKEDLPPLLLEV
PXR_human       372 ITLKSYIECNRPQPAHRFLFLKIMAMLTELRSINAQH-TQRLLRIQDIHPFAT--PLMQEL

VDR_human       422 FGNEIS
VDR_dog             FGNEIS
VDR_mouse           FGNEIS
VDR_chicken         FGNEIS
VDR_xen_laevis      FSDEIP
VDR_salmon          FGSEVS
VDR_stickleback     FGSEVS
VDR_Tetraodon       FGSEVS
VDR_zebrafish       FGSEVS
VDR_medaka          FGSEVS
VDR_lamprey         FGNPTA
PXR_human       429 FGITGS
```
