# Supplementary material for: The evolution of farnesoid X, vitamin D, and pregnane X receptors: insights from the green-spotted pufferfish (Tetraodon nigriviridis) and other non-mammalian species
Source: BMC Biochem. 2011 Feb 3;12:5. doi: 10.1186/1471-2091-12-5 (PMC3042382; doi:10.1186/1471-2091-12-5)
Supplement: Additional file 6 — Data for pharmacophore modeling of Tetraodon nuclear hormone receptors. [file 1471-2091-12-5-S6.PDF]

Additional file 6
Pharmacophore models for *Tetraodon* VDR, PXR, and FXR

Supplemental Table 6a
Pharmacophore data for *Tetraodon* VDR

Compound	FitValue	MaxOmitFeat	Principal	Relative Energy	EC_{50} (μM)	Activity (4 = most active, 0 = inactive)	Efficacy
1,25-Dihydroxyvitamin D_3	5.99912	0	2	15.1648	0.002	4	1.0 (reference)
25-Hydroxyvitamin D_3	4.51151	0	1	4.29164	0.0112	4	1.1
Chenodeoxycholic acid (CDCA)	4.33168	0	0	14.1783		0	
Glyco-CDCA	3.57363	0	0	16.2487		0	
Tauro-CDCA	3.81383	0	0	15.0949		0	
Cholic acid	3.95113	0	0	16.4892		0	
Glycocholic_acid	3.2004	0	0	8.61082		0	
Taurocholic acid	2.43526	0	0	6.21568		0	
Lithocholic acid (LCA)	3.53557	0	0	12.6818	21.6	3	0.12
3-Keto-LCA	2.32824	0	0	14.9259	55	2	0.17
Nor-LCA	4.14119	0	0	11.1148		0	
Iso-LCA	3.53557	0	0	12.6818	70	1	0.04
LCA acetate	3.14115	0	1	19.0789	2.17	3	0.61
5α-Cholan-24-oic acid	2.91095	0	0	10.5032	75	1	0.03
3β-Hydroxy-5α-Cholan-24-oic acid	3.53557	0	0	12.6818		0	
7α-Hydroxy-5β-cholan-24-oic acid	3.47805	0	0	18.7846		0	
12α-Hydroxy-5β-cholan-24-oic acid	3.50707	0	0	10.2479		0	
5α-Cyprinol-27-sulfate	2.54606	0	0	13.3287		0	

Supplemental Table 6b
Pharmacophore data for *Tetraodon* PXR

Compound	FitValue	MaxOmitFeat	Principal	Relative Energy	EC_{50} (μM)	Activity (4 = most active, 0 = inactive)	Efficacy
5α-Androstan-3α-ol	1.64556	0	1	14.6191	6.1	4	1.0 (reference)
5α-Androst-16-en-3α-ol	1.31774	0	1	9.4544	7.4	3	0.58
Androstenedione	2.60049	0	0	7.65671		0	
Cortisone	1.09909	0	1	11.7555	12.2	2	0.19
DHEA_sulfate	2.24588	0	1	4.53119	8.5	3	0.7
Estradiol	1.00728	0	1	3.04545	9.6	3	0.47
5β-Pregnanedione	1.76944	0	2	21.8674	4	4	0.73
Pregnenolone	0.50688	0	2	10.594	3.3	3	0.34
Progesterone	0.99446	0	1	16.984	8.9	3	0.33
T-0901317	3.21084	0	1	14.5108	7.7	2	0.17
Chenodeoxycholic acid (CDCA)	3.82898	0	1	12.8722	15.6	2	0.38
Tauro-CDCA	3.99907	0	2	17.4948	4.8	3	0.59
Lithocholic acid (LCA)	3.9102	0	0	16.9175	81	1	0.1
Tauro-LCA	3.92666	0	1	11.0719	19.1	2	0.29
3-Keto-LCA	3.69163	0	0	14.9259	42.3	2	0.65
Cholic acid	3.8701	0	0	10.556		0	
Taurocholic acid	3.8919	0	0	5.04982		0	
Deoxycholic acid	3.87747	0	1	9.83678	9	2	0.33
5α-Cyprinol-27-sulfate	3.21324	0	1	10.5616	8.2	3	4
4-Amino-butylbenzoate	1.76528	0	0	13.7951	25.6	2	0.88

Supplemental Table 6c
Pharmacophore data for *Tetraodon* FXR

Compound	FitValue	MaxOmitFeat	Principal	Relative Energy	EC_{50} (μM)	Activity (4 = most active, 0 = inactive)	Efficacy
GW4064	5.99936	0	2	6.61784	2.8	4	1.0 (reference)
Lithocholic acid (LCA)	2.38399	0	0	3.91934	20.8	2	0.29
Tauro-LCA	2.33593	0	0	3.42822	29.6	2	0.26
3_Keto_LCA	1.78828	0	1	4.16635	17.9	2	0.61
Chenodeoxycholic acid (CDCA)	1.57484	0	1	14.1783	9	3	0.39
Tauro-CDCA	1.82115	0	1	15.4133	13.2	2	0.15
Deoxycholic acid	1.60702	0	1	1.51702	18.7	2	0.24
5α-Cyprinol-27-sulfate	1.05992	0	1	1.31957	13.7	2	0.15
