# Supplementary material for: The evolution of farnesoid X, vitamin D, and pregnane X receptors: insights from the green-spotted pufferfish (Tetraodon nigriviridis) and other non-mammalian species
Source: BMC Biochem. 2011 Feb 3;12:5. doi: 10.1186/1471-2091-12-5 (PMC3042382; doi:10.1186/1471-2091-12-5)
Supplement: Additional file 7 — Fish phylogeny, bile salt variation, and nuclear hormone receptor ligand specificity. [file 1471-2091-12-5-S7.PDF]

Additional file 7 – Fish phylogeny, bile salt variation, and nuclear hormone receptor ligand specificity

Bile salt variation determined by Hagey et al. (2010) [1] overlaid on the fish tree proposed by Nelson (2006) [2] excluding fish orders whose bile salt profiles are unknown. Alepocephaliformes is used instead of Argentiformes following results of Lavoué et al. (2008) [3], and Syngnathiformes is used instead of Gasterosteiformes following the results of Kawahara et al. (2008) [4]. The major bile salt classes for each fish order are indicated by A (5α-bile alcohols), B (5β-bile alcohols), C (27-carbon or C_{27} bile acids), and D (24-carbon or C_{24} bile acids) as shown in the legend. Some fish orders have multiple bile salt classes.

The nuclear hormone receptors (NHRs) FXR, VDR, and PXR have only been cloned and characterized from a limited number of fish species: green-spotted pufferfish (*Tetraodon nigriviridis*, Tetraodontiformes; FXR, VDR, PXR, characterized in this report), Japanese medaka (*Oryzias latipes*, Beloniformes; FXR, VDR) [5], zebrafish (*Danio rerio*, Cypriniformes; FXR, VDR, PXR) [6, 7], little skate (*Leucoraja erinacea*, Elasmobranchii; FXR) [8], and sea lamprey (*Petromyzon marinus*, Petromyzontiformes; FXR, VDR) [6, 7, 9]. The ligand specificity of the various FXRs, VDRs, and PXRs for bile salts (if it has been characterized) uses the same legend as for the bile salt classes for fish orders. For example, zebrafish (Cypriniformes) has predominantly 5α-bile alcohols for its primary bile salts. Zebrafish FXR and PXR are activated by 5α-bile alcohols (and not by C_{24} or C_{27} bile acids), while the zebrafish VDR is not activated by any bile salts (indicated by 'inactive').

As can be seen in the phylogeny, Cypriniformes is unusual in having fish species with predominantly 5α-bile alcohols as opposed to the C_{24} 5β-bile acids typical of most actinopterygian fish whose bile salts have been characterized so far. In terms of better understanding the evolution of NHR sensitivity to bile salts, characterization of NHRs from Myxiniformes (hagfish), Elasmobranchii (sharks, skates, rays), Chimaeriformes (chimaerae), Ceratodontiformes (lungfish), and Coelacanthiformes (coelacanths) would be of particular interest as they use either 5α- or 5β-bile alcohols and not bile acids as their primary bile salts.

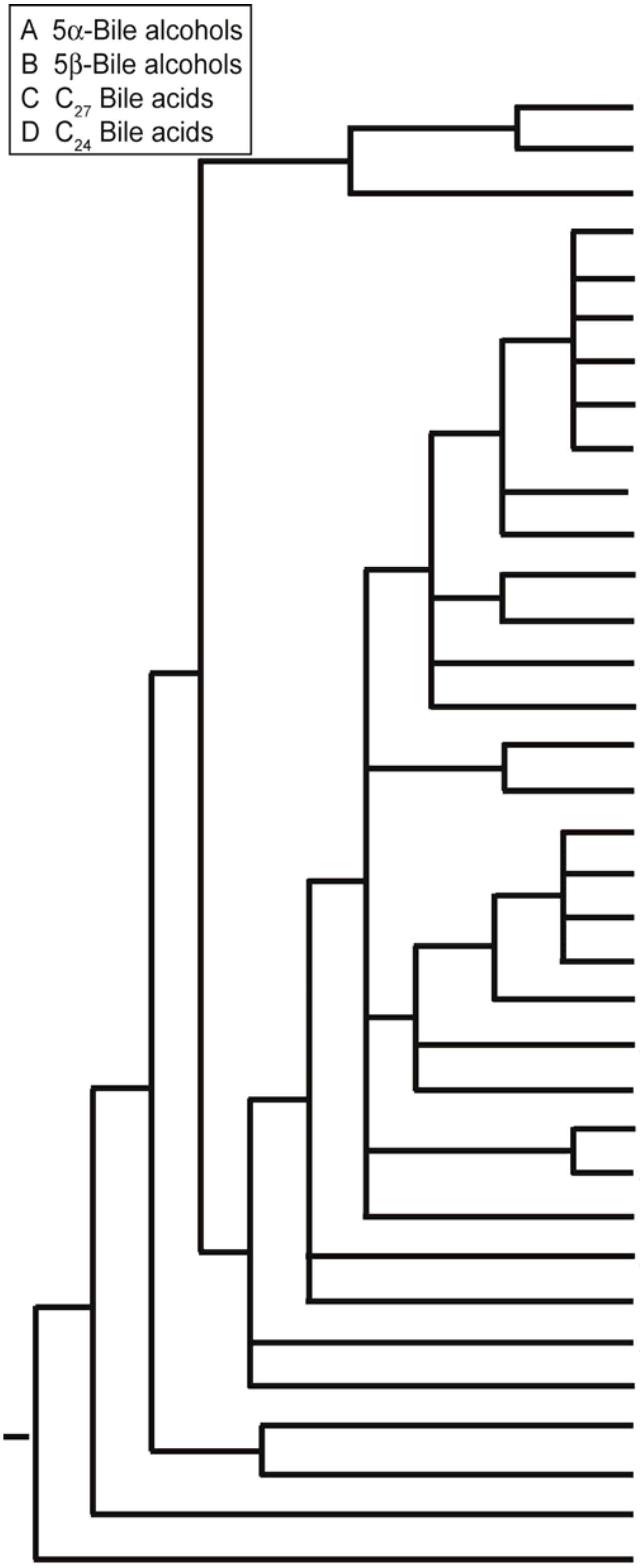

	Bile salt profile	FXR	VDR	PXR
Tetrapoda	Varied	D	D	A,B,D
Ceratodontiformes	A			
Coelacanthiformes	A			
Tetraodontiformes	D	D	D	D
Pleuronectiformes	D			
Perciformes	D, B			
Scorpaeniformes	D			
Syngnathiformes	D, C			
Beryciformes	D			
Beloniformes	D, C	D	D	
Mugiliformes	D			
Lophiiformes	D, C, B			
Gadiformes	D, C			
Aulopiformes	D			
Stomiiformes	D			
Esociformes	D			
Salmoniformes	D			
Gymnotiformes	D			
Siluriformes	D			
Characiformes	D, C			
Cypriniformes	A	A	Inactive	A
Gonorynchiformes	D			
Alepocephaliformes	D, B			
Clupeiformes	D, B			
Saccopharyngiformes	D			
Anguilliformes	D, B			
Osteoglossiformes	D, B			
Amiiformes	D, B			
Lepisosteiformes	D			
Acipenseriformes	D, B			
Polypteriformes	D, B			
Elasmobranchii	B	Inactive		
Chimaeriformes	B			
Petromyzontiformes	A	A	Inactive	
Myxiniformes	A			

References:

1. Hagey LR, Møller PR, Hofmann AF, Krasowski MD: **Diversity of bile salts in fish and amphibians: evolution of a complex biochemical pathway**. *Physiol Biochem Zool* 2010, **83**:308-321.
2. Nelson JS: **Fishes of the World**, 4th edn. Hoboken, NJ: John Wiley and Sons, Inc.; 2006.
3. Lavoué S, Miya M, Poulsen JY, Møller PR, Nishida M: **Monophyly, phylogenetic position and inter-familial relationships of the Alepocephaliformes (Teleostei) based on whole mitogenome sequences**. *Molecular Phylogenetics and Evolution* 2008, **47**:1111-1121.
4. Kawahara R, Miya M, Mabuchi K, Lavoué S, Inoue JG, Satoh TP, Kawaguchi A, Nishida M: **Interrelationships of the 11 gasterosteiform families (sticklebacks, pipefishes, and their relatives): a new perspective based on whole mitogenome sequences from 75 higher teleosts**. *Molecular Phylogenetics and Evolution* 2008, **46**:224-236.
5. Howarth DL, Hagey LR, Law SHW, Ai N, Krasowski MD, Ekins S, Moore JT, Kollitz EM, Hinton DE, Kullman SW: **Two farnesoid X receptor a isoforms in Japanese medaka (*Orzias latipes*) are differentially activated in vitro**. *Aquat Toxicol* 2010, **98**:245-255.
6. Krasowski MD, Yasuda K, Hagey LR, Schuetz EG: **Evolution of the pregnane X receptor: adaptation to cross-species differences in biliary bile salts**. *Mol Endocrinol* 2005, **19**:1720-1739.
7. Reschly EJ, Ai N, Ekins S, Welsh WJ, Hagey LR, Hofmann AF, Krasowski MD: **Evolution of the bile salt nuclear receptor FXR in vertebrates**. *J Lipid Res* 2008, **49**:1577-1587.
8. Cai SY, Xiong L, Wray CG, Ballatori N, Boyer JL: **The farnesoid X receptor, FXRa/NR1H4, acquired ligand specificity for bile salts late in vertebrate evolution**. *Am J Physiol Regul Integr Comp Physiol* 2007, **293**:R1400-R1409.
9. Krasowski MD, Yasuda K, Hagey LR, Schuetz EG: **Evolutionary selection across the nuclear hormone receptor superfamily with a focus on the NR1I subfamily (vitamin D, pregnane X, and constitutive androstane receptors)**. *Nucl Recept* 2005, **3**:2.
